# 3D Printing of Tough Hydrogel Scaffolds with Functional Surface Structures for Tissue Regeneration

**DOI:** 10.1007/s40820-024-01524-z

**Published:** 2024-09-29

**Authors:** Ke Yao, Gaoying Hong, Ximin Yuan, Weicheng Kong, Pengcheng Xia, Yuanrong Li, Yuewei Chen, Nian Liu, Jing He, Jue Shi, Zihe Hu, Yanyan Zhou, Zhijian Xie, Yong He

**Affiliations:** 1 State Key Laboratory of Fluid Power and Mechatronic Systems, School of Mechanical Engineering, Zhejiang University, Hangzhou, 310027 People’s Republic of China; 2 Liangzhu Laboratory, Zhejiang University, Hangzhou, 310027 People’s Republic of China; 3 Key Laboratory of 3D Printing Process and Equipment of Zhejiang Province, College of Mechanical Engineering, Zhejiang University, Hangzhou, 310027 People’s Republic of China; 4 Stomatology Hospital, School of Stomatology, Zhejiang University School of Medicine, Hangzhou, 310027 People’s Republic of China; 5 State Key Laboratory of Advanced Welding and Joining, Harbin Institute of Technology, Harbin, 150001 People’s Republic of China; 6 Institute of Digital Medicine, Nanjing First Hospital, Nanjing Medical University, Nanjing, 210006 People’s Republic of China

**Keywords:** 3D printing, Tough hydrogel scaffold, Functional surface structure, Tissue regeneration, Biomaterials

## Abstract

Hydrogel scaffolds have numerous potential applications in the tissue engineering field. However, tough hydrogel scaffolds implanted in *vivo* are seldom reported because it is difficult to balance biocompatibility and high mechanical properties. Inspired by Chinese ramen, we propose a universal fabricating method (printing-P, training-T, cross-linking-C, PTC & PCT) for tough hydrogel scaffolds to fill this gap. First, 3D printing fabricates a hydrogel scaffold with desired structures (P). Then, the scaffold could have extraordinarily high mechanical properties and functional surface structure by cycle mechanical training with salting-out assistance (T). Finally, the training results are fixed by photo-cross-linking processing (C). The tough gelatin hydrogel scaffolds exhibit excellent tensile strength of 6.66 MPa (622-fold untreated) and have excellent biocompatibility. Furthermore, this scaffold possesses functional surface structures from nanometer to micron to millimeter, which can efficiently induce directional cell growth. Interestingly, this strategy can produce bionic human tissue with mechanical properties of 10 kPa-10 MPa by changing the type of salt, and many hydrogels, such as gelatin and silk, could be improved with PTC or PCT strategies. Animal experiments show that this scaffold can effectively promote the new generation of muscle fibers, blood vessels, and nerves within 4 weeks, prompting the rapid regeneration of large-volume muscle loss injuries.

**Supplementary Information:**

The online version contains supplementary material available at 10.1007/s40820-024-01524-z.

## Highlights

- We propose the novel concept of a tough hydrogel scaffold within the realm of tissue engineering. This scaffold combines exceptional strength (6.66 MPa), customization capabilities, and superior biocompatibility in a manner not previously achieved in existing research.
- These tough hydrogel scaffolds possess functional surface structures and can effectively enhance cell-guided growth and prompt regeneration of muscle tissue in vivo.
- This is a universal manufacturing method for tough hydrogel scaffolds in tissue engineering.

**Supplementary Information:**

The online version contains supplementary material available at 10.1007/s40820-024-01524-z.

## Graphical abstract

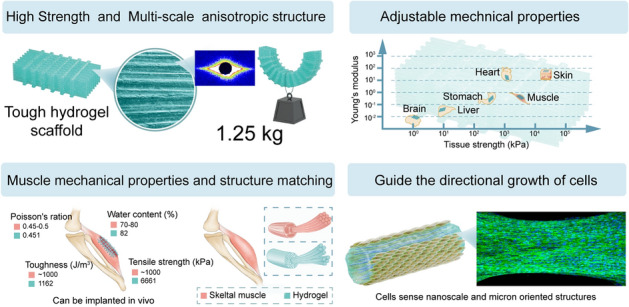

## Introduction

Hydrogel scaffolds have been widely used in tissue regeneration over the past few decades due to their high-water content and customized structure, and are commonly used in skin repair [1, 2], drug delivery [3], cartilage regeneration [4–6], blood vessel maturation [7, 8], and other applications. An ideal implanted material would closely resemble the mechanical characteristics of the target tissue because different natural tissues react differently to mechanical forces [9]. Naturally derived hydrogels, e.g., gelatin, collagen, chitosan, and hyaluropinic acid (HA), are widely used in biological applications because of their biological function. However, the mechanical properties of gelatin, alginate and HA are less than 10 kPa [10–12] and that of chitosan is less than 20 kPa [13]. Due to these poor mechanical properties of biohydrogels, applications are limited in repairing strong soft tissues such as muscles and tendons [9, 14].

Many reports have been dedicated to improving the mechanical properties of hydrogels, such as non-covalently reinforced chemically cross-linked hydrogel scaffolds [15], interpenetrating network hydrogel scaffolds [16, 17], mechanically stretched hydrogel scaffolds [18], and freeze cast hydrogel [19, 20]. The strong hydrogen bond interaction and nanochannel confinement of the hydrogel polymer segments prevent crack propagation and alleviate stress concentration at the crack tip, achieving rapid self-reinforcement [21]. Another strategy is freeze casting and salting out, which can produce highly anisotropic hydrogels with micron-sized honeycomb pore walls and interwoven nanofiber networks with properties similar to those of real tendons [20]. Repeated mechanical loading causes the physically cross-linked hydrogel to rearrange along the loading direction, resulting in directionally aligned nanofibers with inherently improved mechanical properties [18, 22]. A kind of tough adhesive hydrogel was developed by combining imidazole-containing polyaspartamide and an energy-dissipative alginate–polyacrylamide double network. After linear stretching and secondary cross-linking fixation, this hydrogel obtained anisotropic structure and high mechanical properties [23]. Although tough hydrogel can be developed by various techniques, most of these methods are incapable of handling biohydrogels, or are not conducive to customizing hydrogel structures, a very important task in tissue regeneration.

Few reports have addressed the above challenge. 3D-printable and highly stretchable tough hydrogel by combining polyethylene glycol and sodium alginate was reported by Zhao [24]. Together, these form a tougher hydrogel than natural cartilage, enabling cells to maintain high viability after 7 days of culture. A biodegradable, high-strength hydrogel reinforced by supramolecular polymers was fabricated through the photoinitiated polymerization and introduction of hydrogen bonding to strengthen PACG [25]. Although the mechanical properties of biological grade hydrogels could be improved, the complex ingredients (inconsistent with tissue composition) and cumbersome preparation process limit their application. Therefore, the existing tissue engineering repair methods can be greatly expanded by a strategy that can easily manufacture tough hydrogel scaffolds with customized structure that resemble natural tissue components, which this article proposes.

**Fig. 1:**
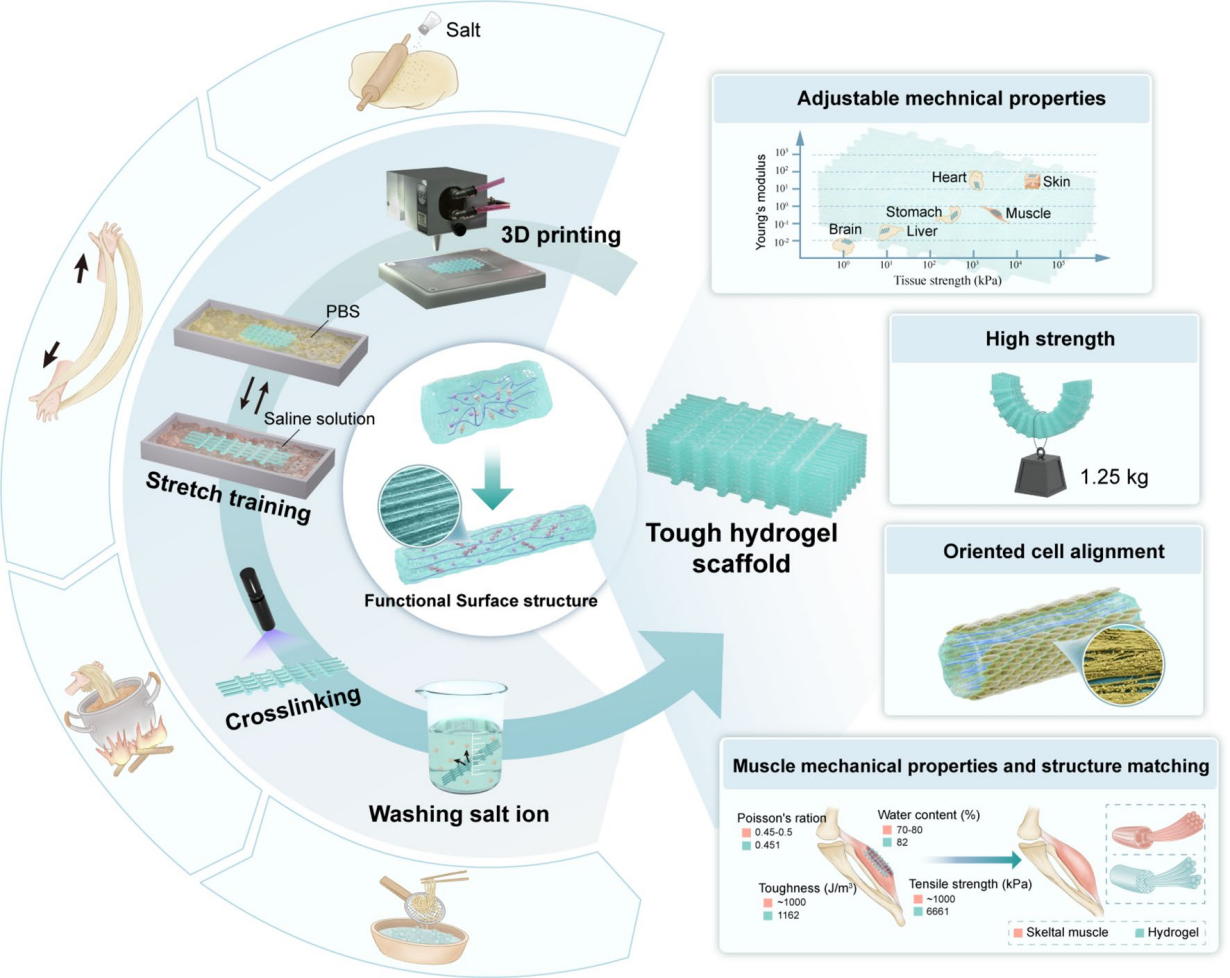
Preparation method and characteristics of tough hydrogel scaffold

Herein, inspired by Chinese ramen, we propose a novel and convenient strategy to address the above-mentioned problem that biohydrogels have poor mechanical properties and are difficult to tailor to individual structures. First, 3D printing is implemented to prepare the initial hydrogel scaffold with the desired structure. Then, cyclic mechanical training and salting-out assistance are performed to ascribe the scaffold with extremely high mechanical properties. Finally, the training results are fixed by photo-cross-linking (Fig. 1). The hydrogel scaffold demonstrates a remarkable tensile strength of 6.66 MPa, surpassing that of untreated materials by 622 times. Additionally, it exhibits high toughness measuring up to 1162.71 kJ m^−3^. Interestingly, the scaffold has a functional surface structure, including micron-scale oriented fibers and nanoscale oriented molecular chain networks, which can effectively induce directional cell growth. In addition, this strategy can produce biomimetic human tissue scaffolds with mechanical properties of 10 kPa-10 MPa by changing the type of salt. We demonstrate that many hydrogels, such as gelatin and silk, can be improved by PTC or PCT strategies. We take gelatin-based hydrogel as an example and verify its feasibility for rapidly repairing large tissue muscle loss. This strategy provides a novel universal approach to fabricating high-strength hydrogel scaffolds and is expected to become a common method in tissue regeneration engineering.

## Experimental Section

### Materials

Phosphate-buffered saline (PBS) was acquired from Zhejiang Jinuo Biomedical Technology Co., Ltd. Lithium phenyl-2,4,6-trimethylbenzoylphosphinate (LAP) and GelMA (EFL-GelMA-30) was bought from Yongqinquan lntelligent Equipment Co., Ltd, Suzhou, China; ammonium sulfate ((NH_4_)_2_SO_4_, AR, 99%) was purchased from Macklin. Polyether F127 Diacrylate(F127DA), methacrylated hyaluronic acid and other hydrogels were obtained from Yongqinquan lntelligent Equipment Co., Ltd, Suzhou, China; polydimethylsiloxane (PDMS, Dow Corning) was from Dow Corning Ethanol (95%) was acquired from Shanghai Lanqing Industrial Co., Ltd. Potassium chloride, sodium chloride, sodium carbonate and other salts were bought from Dengfeng Fine Chemicals, China. Type-B gelatin, methacrylic anhydride (MA, ≥ 94%), sodium hydroxide (NaOH, bioXtra, ≥ 98%), calcein-AM (bioreagent, ≥ 95.0%) and propidium iodide (PI, ≥ 94%) were purchased from Sigma-Aldrich; fetal bovine serum (FBS, Biological Industries, 04-001-1A) was acquired from Hyclone, Hong Kong. Deionized (DI) water was prepared by using a laboratory water purification system.

### Fabrication of Hydrogel Solution

GelMA hydrogels are obtained from Yongqinquan lntelligent Equipment Co., Ltd, Suzhou, China. First, prepare a 0.3% LAP solution using PBS. Next, add 2 g of hydrogel to a pointed bottom centrifuge tube (50 mL), take 25 mL of LAP solution and pour it into it, then place the centrifuge tube in a 50 °C constant temperature water bath until completely dissolved, then remove it into 10 cc dispensing syringe and store it in 20–30 °C oven for future printing use.

### Rheological Characterization

The hydrogels were characterized by rheology using an Anton Paar MCR302 rotational rheometer with a 20-mm-diameter parallel plate. Testing was performed at 37 °C and room temperature. A solvent trap was used to ensure temperature stability. A 0.5 mL volume of hydrogel was incubated in 37 °C water bath before being transferred to the rheometer surface to take measurements. The shear thinning behavior was investigated by measuring shear viscosity in continuous flow at a ramped shear rate from 5 to 50 s^−1^. Strain sweeps ranging from 5 to 50 rad s^−1^ at a 1% amplitude. To demonstrate the low-temperature printability of hydrogels, the G’ and G’’ values were measured from 5–45 °C. To test the photosensitive properties of GelMA, an in situ photorheology was performed with a 5 rad s^−1^ and 1% strain. G’ and G’’ for GelMA were measured at a frequency of 1 Hz.

A temperature sweep rheometer (MCR102, Anton Paar, Austria) was used to test GelMA’s sensitivity to temperature. The GelMA samples were cooled from 45 to 5 °C at a rate of 2 °C min^−1^ with a 5 rad s^−1^ and 1% strain after being equilibrated at 45 °C. G' at 5 °C and the various sample gel-sol transition temperatures were examined. A 5 rad s^−1^ and 1% strain in situ photorheology was carried out to investigate GelMA’s photosensitive characteristics. At a frequency of 1 Hz, the GelMA parameters G' and G'' were measured.

### Hydrogel Scaffold Printing

The G code is generated by the EFL_PotatoE software developed by Yongqinquan lntelligent Equipment Co., Ltd, Suzhou, China. Hydrogel scaffolds were printed using the BP6601Pro bioprinter developed by Yongqinquan lntelligent Equipment Co., Ltd, Suzhou, China. The printing barrel temperature is set to 20–30 °C, and printing platform temperature is set to 10–15 °C. The printed hydrogel scaffold is stored in a refrigerator at 2–8 °C for subsequent strengthening use.

### Hydrogel Strengthening

Customize two rectangular acrylic boxes (without lids) to store 50% ammonium sulfate solution and PBS solution, respectively. Design and build automatic circuit training and strengthening equipment. Fix the uncross-linked hydrogel on the stretching fixture, cyclically stretch it for a certain number of times in ammonium sulfate solution, and place it in PBS solution for a period. According to this cycle for a certain number of times, and use a 405-nm wavelength UV curing lamp to cure the trained hydrogel, a super-strong hydrogel can be obtained.

### Simulation Analysis Method

The process is simulated by using the DMol3 module in materials studio. The atomic structure of the hydrogel is established in the module of DMol3. In the generalized gradient approximation (GGA), all density functional theory (DFT) calculations are carried out by using PBE formula, and Basisset is set to DNP4.4. Set the convergence error of the calculation, the energy: 2 × 10^–5^ Ha, the maximum convergence force: 0.004 Ha Å^−1^, the maximum displacement: 0.005 Å, and the convergence steps 500.

### Mechanical Characterization

The test was produced into the shape of a dumbbell (45 mm in length, 10 mm in breadth, and 2 mm in thickness), mounted on an electronic universal testing machine (UTM2102, Shenzhen Sun Technology Co., Ltd.), and put through a tensile test at 5 mm per minute while under displacement control.

The toughness of a hydrogel scaffold was calculated by integration area under stress–strain curves of unnotched specimen from original point to critical strain point (${\varepsilon }_{c}$) of notched specimen, as formula:

1 $$Toughness={\int }_{0}^{{\varepsilon }_{c}}\sigma d\varepsilon$$

where the normal stress ($\sigma$) was the recorded forcedivided by the initial cross-sectional area and the strain ($\varepsilon$) was measured from themeasuring distance divided by the initial distance.

### Water Content Measurement

The water content of the hydrogel was measured by comparing the weights before and after high-temperature drying (60 °C, 24 h). The weight before (${m}_{0}$) and after (${m}_{a}$) high-temperature drying. The water content was obtained as:

2 $$water\; content=\frac{{m}_{0}-{m}_{a}}{{m}_{0}}\times 100\%$$

### Hear Thinning Analysis

GelMA solution was tested in a rheologic shear rate sweep test (MCR102, Anton Paar, Austria) with a shear rate range of 5–50 rad s^−1^ and a printing window of 24–26 °C to demonstrate the shear thinning property of GelMA.

#### Swelling Testing

By employing a common liquid scenario (phosphate buffer solution, PBS), the weighing method was employed to assess the swelling capabilities of GelMA. The previously mentioned printing method was used to create hydrogel scaffolds. Samples were then obtained at 0, 2, 4, 6, 12, and 24 h after being rinsed with 2 mL of sterile PBS at 37 °C. The extra liquid was wiped away with wax paper. To determine the swollen weight (${w}_{a}$), the samples were weighed after that. The initial hydrogel weight is ${w}_{0}$.

3 $$Swelling\; Rate=\frac{{w}_{a}-{w}_{0}}{{w}_{0}}\times 100\%$$

#### Degradation Testing

Accelerating Hydrogel Scaffold Degradation Using Type II collagenase (Biofroxx, Guangzhou, China). By employing a common liquid scenario (phosphate buffer solution, PBS), the weighing method was employed to assess the swelling capabilities of GelMA. The previously mentioned printing method was used to prepare hydrogel scaffolds. After being cleaned in 2 mL of sterile PBS at 37 °C for 24 h, the samples were weighed to determine equilibrium swelling. The samples were then put into 2 U mL^−1^ of type II collagenase in PBS. At 0, 0.5, 1, 2, 3, and 4 h, they were collected. The sample's weight was measured as *W*_d_ after the surface moisture was wiped off. It is noted that the initial hydrogel mass is *W*_1_. The following equation was used to calculate the mass degradation rate.

4 $$Degradation\; Rate=\frac{{w}_{1}-{w}_{d}}{{w}_{1}}\times 100\%$$

In *vivo*, each group of hydrogel stents were implanted subcutaneously in mice and then sampled for 7 days.

#### Biocompatibility Analysis

C2C12 multiple myoblasts were cultured in DMEM containing 10% Fetal bovine serum (Gibco, USA) and 1% penicillin–streptomycin solution (Sigma, USA). According to ISO 10993–5, the hydrogel scaffolds were immersed in the culture medium for 24 h with extraction ratio (surface area/volume) at 3 cm^2^ mL^−1^ to obtain the extraction. The viability of C2C12 myoblasts was evaluated with a Calcein-AM/propidium iodide (PI) Live-Dead Cell Staining Kit. According to manufacturer's requirements, cells were first incubated in Calcein-AM staining solution for 30 min under dark conditions and then incubated in PI staining solution for 15 min. Then, a fluorescence microscope (Zeiss) was employed to take fluorescence photographs. To detect cell proliferation ability, culture medium was added with 10% Cell Counting Kit solution (CCK-8). After an incubation of 40 min, the absorbance at 450 nm was measured using a Microplate Reader (Perkin-Elmer, USA).

#### Hemolysis Rate Assay

In vitro hemolysis rate assay was used to assess the blood compatibility of the hydrogel scaffolds. Firstly, fresh anticoagulated rabbit whole blood (with sodium citrate) was diluted with 0.9% normal saline. Then, the hydrogel scaffolds were added to diluted blood. The diluted blood added with normal saline served as a negative control and ddH_2_O as a positive control. After culturing at 37 °C for 30 min, 200 μL diluted blood was drawn into a centrifuge tube and further cultured for 1 h. The tubes were then centrifuged at 800 g for 5 min. The OD values of supernatant in each group were measured at 540 nm. The hemolysis rate was calculated using the equation:

5 $${\text{Hemolysis rate }}\left( {\text{\% }} \right) = \frac{{{\text{OD}}_{{{\text{sample}}}} { } - {\text{ OD}}_{{{\text{negative}}}} }}{{{\text{OD}}_{{{\text{positive}}}} { } - {\text{ OD}}_{{{\text{negative}}}} }} \times 100$$

#### Cell Adhesion and Oriented Growth Analysis

The hydrogel scaffolds of each group were placed in a 48-well plate, and then 8×10^3^ cells per well were seeded on the hydrogel surface. After culturing for 24 h, C2C12 were fixed with 4% Paraformaldehyde for 20 min and penetrated with 0.5% Triton X-100. Then, Phalloidin (Cytoskeleton Inc, USA) was used to stain cytoskeleton for 40 min and DAPI (Beyotime, China) was applied to stain cell nuclei for 5-min under dark condition. The laser confocal scanning microscope (CLSM, Leica Microsystems, German) was used to observed cell morphology and adhesion.

#### Volumetric Muscle Loss Injury and Bioconstruct Implantation

All the procedures involving animals were performed in accordance to laboratory animal ethics requirements and were approved by the Ethics Committee for Laboratory Animal Welfare Ethics Committee of Zhejiang Laboratory Animal Center (approval number: ZJCLA-IACUC-20010310). C57BL/6 mice (8–10 weeks of age) were purchased from Zhejiang Experimental Animal Center and used for the study. Volumetric muscle loss (VML) injury was created on the tibialis anterior muscle as follows: Under general anesthesia, depilation of legs was performed using a clipper and razor. After disinfection and local anesthesia, a skin incision below the knee joint was made to expose the anterior tibial muscle. A defect approximately 2 mm × 1 mm × 7 mm was manually resected in the tibialis anterior using a scalpel. For the no treatment group (NT, *n* = 4), the injury was left without any treatment. For the control group (Ctr, *n* = 4), the hydrogels without training were implanted at the injury sites. For the experiment group (PTC, *n *= 4), the training hydrogels were implanted at the injury sites. Then, the fascia was sutured to keep the scaffold in place, and the skin was stapled closed. The mice were euthanized on the 14th and 28th post-surgery day, and the harvested tissues were used for histological analysis.

#### Histological and Immunohistochemical Analysis

The harvested muscle was fixed in the muscle specific fixative (Servicebio, China) for 24 h and the cut into 5 μm cross sections. Hematoxylin and eosin (H&E) staining and Masson trichrome staining were performed on the sections to evaluate muscle fiber regeneration and collagen deposition. The digital slicing scanner (Olympus VS200, Japan) was used to obtain muscle cross-section images. The number of myofibers with centrally located nuclei and the area of collagen deposition were quantified by Image J.

For immunohistochemical staining, the sections were firstly incubated at 4 °C overnight with the following primary antibodies: anti-myosin heavy chain (MHC, Abcam, USA), anti-nicotinic acetylcholine receptor (AchR, Abcam, USA), α-smooth muscle actin (α-SMA, Proteintech, China), and CD31 (affinity, China). Then the sections were washed with PBS and incubated with the secondary antibodies. The area of the α-SMA-positive, CD31-positive, AchR-positive, and the myofiber cross-sectional Feret diameter were quantified by Image J.

#### Histological and Immunohistochemical Analysis

The animal experiments were reviewed and approved by the Experimental Animal Welfare Ethics Committee of Zhejiang Experimental Animal Center.

Approval number: ZJCLA-IACUC-20010310.

## Results and Discussion

### Tough 3D-Printed Hydrogel Manufacturing Method

**Fig. 2:**
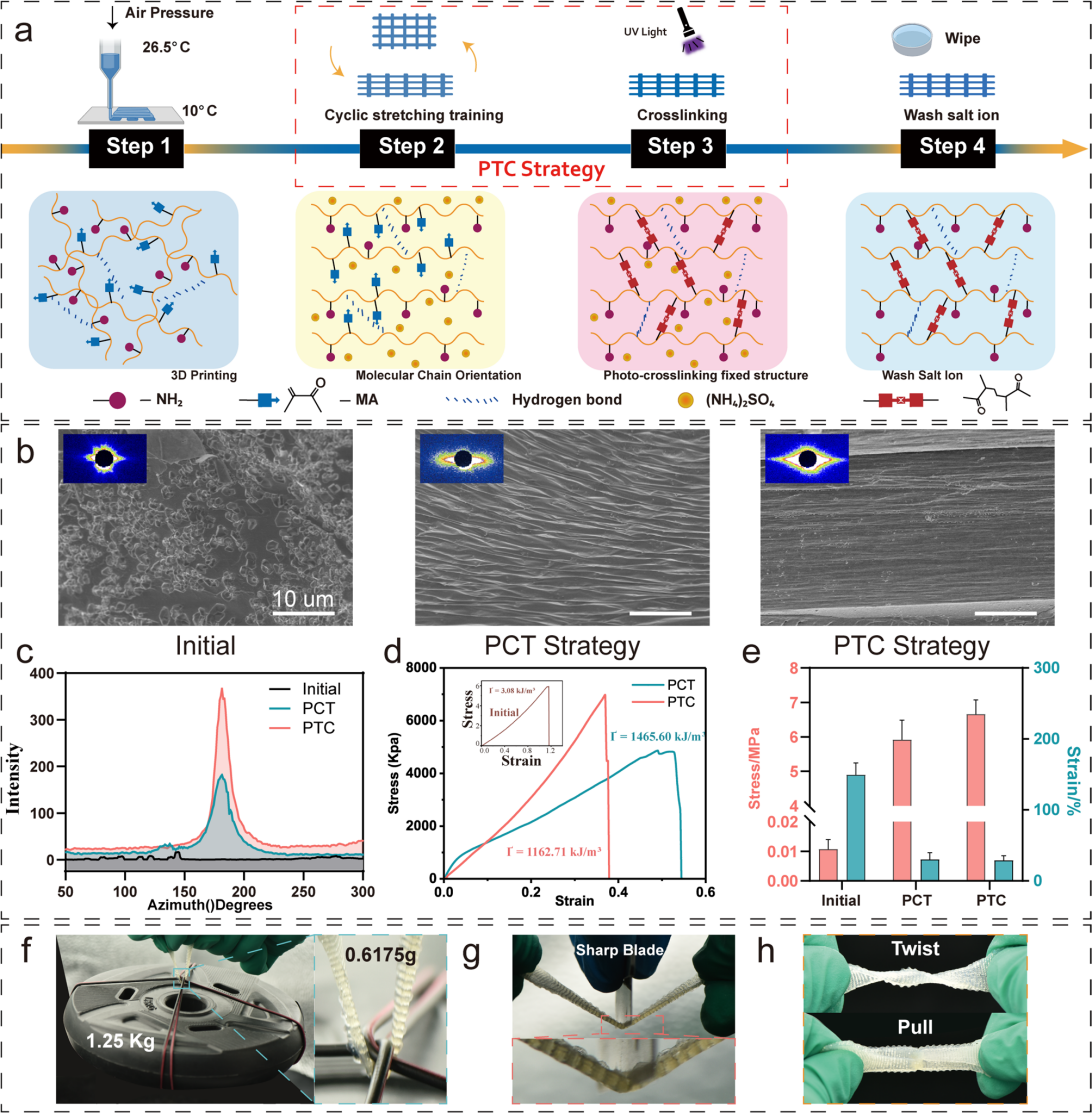
Schematic diagram and results of tough hydrogel preparation. **a** Schematic diagram of PTC hydrogel preparation. **b** Tough hydrogel with functional structure. scale bar = 10 μm **c** Tough hydrogel molecular chain orientation. **d** Tough hydrogel stress–strain curve. **e** Tough hydrogel stress strain histogram. **f** Tough hydrogel for weightlifting. **g** Tough hydrogel for withstanding sharper blades. **h** Tough hydrogel for twist and pull

According to the Hofmeister effect, different ions have varying capacity to precipitate protein, which can be used to modify the protein aggregation states by the straightforward addition of particular ions [26]. Modulus-adjusted structures could be created from the same protein composition with the aid of certain ions. While encouraging molecular concentration, directional stretch gives hydrogels a functional surface structure at larger (micrometer-millimeter) scales [20]. Here, we suggest combining molecular and structural engineering methods to create hydrogels. We first integrate directed stretch and salting-out treatment, which work together to form hydrogel structures on various length scales from the molecular level to the millimeter scale, then use the photo-cross-linking method to fix the molecular chain structure (Fig. 2a). During training, we place the hydrogel in an ammonium sulfate solution for repeated stretching. Phase separation occurs through salting out, and ammonium sulfate replaces the position of water molecules, forming a directional microstructure on the surface and interior of the hydrogel scaffold, promoting the orderly arrangement of hydrogel molecular chains [27]. The hydrogel is then placed in a PBS solution to release the pre-stretch, and ammonium sulfate is released from the hydrogel to ensure the biocompatibility of the hydrogel scaffold. After several training rounds, we construct strong, tough and stretchable hydrogels (denoted as PTC hydrogels) with functional surface structures.

Similarly, as a general method for manufacturing tough hydrogels, the method of stretching training combined with salting out is also suitable for 3D-printed hydrogel scaffolds that cannot be photo-cross-linked or require photo-cross-linking before they can be formed (denoted as PCT hydrogels) (Fig. S1). Our group has reported a similar manufacturing method in previous articles and successfully used it in tendon repair [27].

Because NH_4_^+^ and SO_4_^2−^ are strongly kosmotropic ions in the Hofmeister series and create a "salting-out" effect on proteins, ammonium sulfate is frequently employed to precipitate proteins. The Hofmeister series is an order of ions that have the ability to alter the solubility of proteins [28]. The tough hydrogel obtained after the above-mentioned directional stretching and salting-out treatment has a functional surface structure at the micro-level compared to the initial hydrogel (Fig. 2b). It can also be seen through SAXS testing that the tough hydrogel obtained after training has an obvious directional structure not only at the micro-level but also at the molecular level (Fig. 2b, c).

More importantly, the tough hydrogel prepared with the assistance of cyclic stretching and salting out not only effectively improves the degree of molecular chain orientation but also enhances the tensile breaking strength and toughness of the hydrogel scaffold. Our PTC strategy can increase the tensile strength of ordinary hydrogel by 622-fold, which is 6.66 MPa, and toughness of 1162.71 kJ m^−3^. Meanwhile, the PCT strategy can increase the strength of ordinary hydrogel by 553-fold, and tensile strength reaches 5.92 MPa, toughness of 1465.60 kJ m^−3^ (Fig. 2d, e). The reason why the strengthening effect of C-T hydrogel is not as good as that of PTC hydrogel is that after training, the PTC hydrogel effectively fixes the oriented molecular chain structure generated by training through photo-cross-linking, which hinders the crack expansion during tensile fracture. However, because the results of stretching training are fixed by photo-cross-linking, the pre-stretched molecular chains remain highly ordered, so the fracture strain of PTC hydrogel is slightly lower than that of PCT hydrogel, this also causes its toughness to be lower than that of PCT hydrogel. PTC tough hydrogel scaffold can withstand a weight of 1.25 kg (2024-fold of its own weight) (Fig. 2f). Although the 3D-printed hydrogel is a porous structure [14], this tough hydrogel can withstand the action of sharper blades without breaking after being trained by stretching and salting out (Fig. 2g). Furthermore, this tough hydrogel can resist twisting and pulling forces without breaking (Fig. 2h).

### Strengthening Mechanism of the Tough Hydrogel Scaffold

We first investigated the strengthening mechanism of hydrogel scaffolds during stretching training in an ammonium sulfate solution.

Gelatin-methacrylate (GelMA) is a biocompatible and photocurable hydrogel comprising gelatin and methacrylate (MA) [29], which was first synthesized by Bulcke et al. in 2000 [30]. Gelatin, which constitutes over 99% of the chemical composition of GelMA, is produced by hydrolyzing collagen and has a biocompatibility that is similar to that of the extracellular matrix (ECM) [31]. The gelatin molecules are made cross-linkable by adding methacryloyl, a functional group with double bonds and the second key component of GelMA, increasing its formability [14].

A large amount of gelatin obtained from nature is still essentially an incomplete degradation product of the unrequited love molecule obtained after the destruction of the collagen triple helix [32]. Gelatins from sources contain approximately 23% hydroxyproline and proline [33]. Overall, gelatin has about 19 amino acids, the largest of which being glycine, accounting for about 32% [34]. Gelatin usually has excellent thermal stability. A dynamic change in temperature can reflect the thermal denaturation of protein. The hydrogen bonds formed in the condensed state mainly include hydrogen bonds formed by glycine residues, hydroxyproline residues and hydrogen bonds formed between water molecules and molecular chains (Fig. S2). With increasing heating temperature, energy is absorbed, and hydrogen bonds in the gelatin molecules break, resulting in the change of the state of the gelatin structure from an ordered to a disordered one and the unfolding of protein molecules [35]. Gelatin molecules become GelMA after adding methacryloyl groups. GelMA retains the similar properties of gelatin and ECM components and is temperature sensitive and shear thinning. During the 3D printing process, as the temperature decreases, the number of triple helix structures and hydrogen bonds in the hot GelMA solution increases, and the gel gradually transforms into a gel state, a process called physical cross-linking [29]. (Fig. S2).

In this paper, GelMA is established as a long polypeptide chain composed of 13 amino acid fragments, with its component unit of NH_2_–CH(R)–COOH (Fig. S3). The R group in the side chain determines its physical and chemical properties and spatial structure [36]. Generally, the side chain R affecting the mechanical properties must contain –NH_2_, –OH, and –COOH functional groups, and –NH_2_ is occupied during cross-linking [37].

**Fig. 3:**
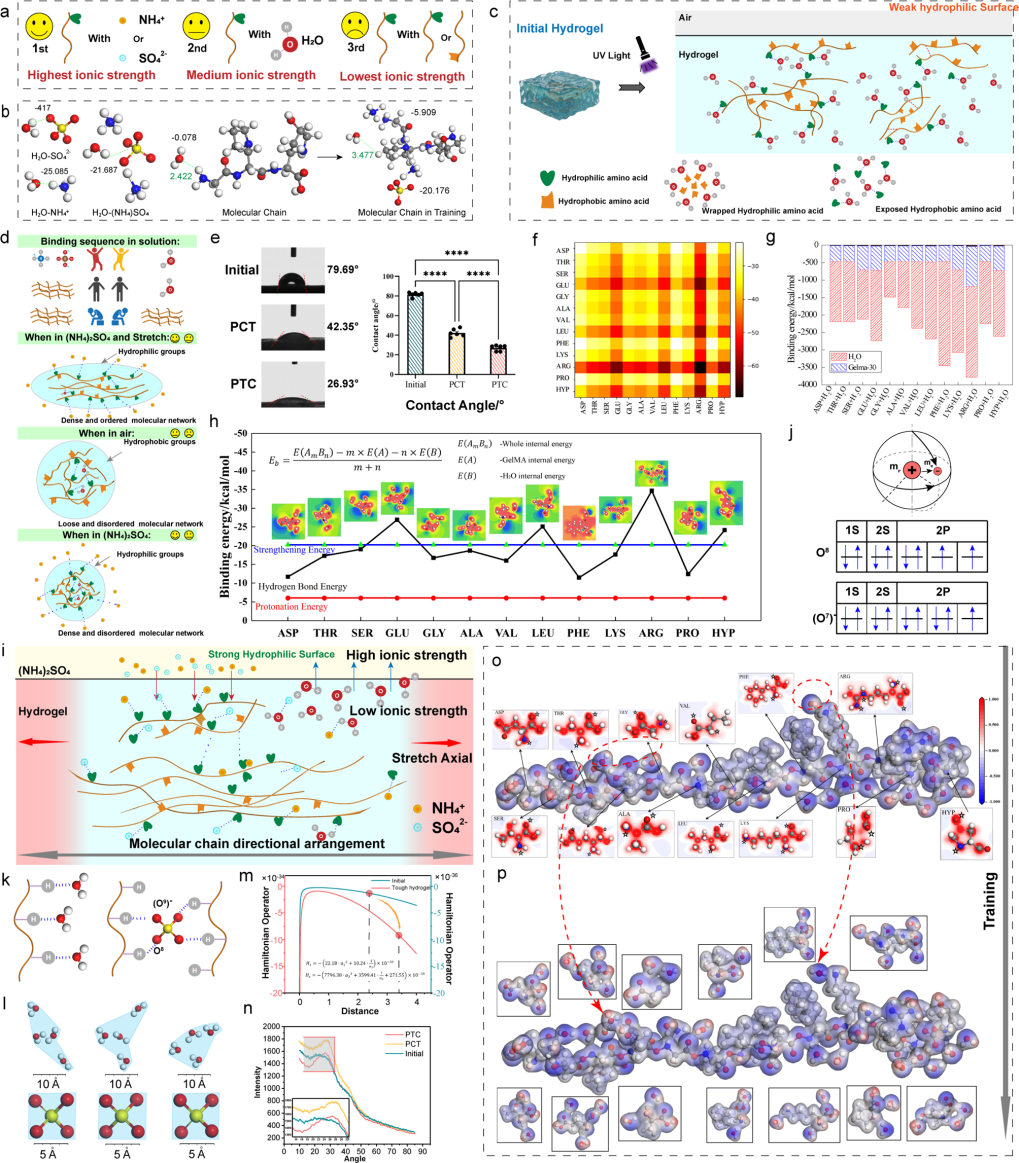
Strengthening mechanism of tough hydrogel scaffold. **a** Hydrogen bonding sequence. **b** Binding energy and bond length in the process of training. **c** Initial hydrogel state after cross-linking. **d** Binding sequence of Amino acids in solution. **e** Contact angle. **f** Amino acid fragment internal energy. **g** The binding energy of amino acid fragments to water molecules. **h** Charge distribution states of 13 amino acid fragments. **i** Schematic diagram of molecular changes. **j** Electron distribution of hydrogen and oxygen atoms. **k** Hydrogen bonding sites for H_2_O and SO_4_^2−^. **l** Volume of SO_4_^2−^ and H_2_O in hydrogen bonding. **m** Enhance model curve. **n** XRD spectra of hydrogel scaffold. **o** Charge distribution states of amino acid fragments. **p** Hydrogel molecular chain after training

Interactions between charged residues can provide specificity since charges can be positive or negative, wherein opposite charges attract and identical charges repel [36]. Therefore, during the training process, the binding order of the hydrogel molecular chain is related to the charge intensity of the bound substance. (NH_4_)_2_SO_4_ has the highest ionic strength and first binds to the hydrophilic molecular chain. The water molecule has medium ionic strength and binds with the hydrophilic peptide chain thereafter. Finally, bonding occurs between molecular chains. The binding energies of ammonium ion, sulfate ion, ammonium sulfate and water are all higher than the binding energies of water molecules and molecular chains. The combination sequence of each component is shown in Fig. 3a. Via calculation, the binding energies of SO_4_^2−^, NH_4_^+^, and (NH_4_)_2_SO_4_ with water are − 417, − 25.085, and − 21.687, respectively, which are higher than those of hydrophilic molecular chains with other particles (− 5.909 and − 20.176) (Fig. 3b).

In air, water molecules preferentially bind to hydrophilic peptide chains, and hydrophobic peptide chains are exposed on the surface of the hydrogel scaffold. Therefore, after direct photo-cross-linking without intensive training, water molecules wrap the hydrophobic peptide chains, and the hydrophilic peptide chains are evenly mixed with water molecules in the scaffold, while more hydrophobic molecular chains accumulate on the surface of the hydrogel (Fig. 3c). In (NH_4_)_2_SO_4_ solution, due to the strong ionic interaction, SO_4_^2−^ and NH_4_^+^ preferentially combine with water molecules in the scaffold, and the hydrogel network becomes dense, which helps to improve the mechanical properties of the scaffold. Moreover, the hydrophilic peptide chains are gradually exposed to the surface of the hydrogel scaffold, which helps to improve the surface hydrophilicity of the hydrogel. As shown in Fig. 3d, e, the average contact angle of the untrained initial hydrogel is 79.69°, the contact angle of the hydrogel trained by the PCT strategy is 42.35°, and the contact angle of the hydrogel trained by the PTC strategy is 26.93°. The surface hydrophilicity of tough hydrogels after training significantly improves, which is more conducive to cell adhesion and proliferation.

We further verified the above notion through molecular dynamics analysis. We first calculated the charge distribution state of 13 amino acid fragments (Fig. 3f) to determine the internal energy of the short peptide chain formed by the combination of each amino acid. Next, the binding energy between 13 amino acid fragments and water molecules was calculated using the first principles (Fig. 3g), which verified the mechanism of GelMA reinforcement.

The density functional theory can be used to calculate the distribution of charges around amino acid fragments and further analyze the principles of bond breaking and bonding during the training process [38]. The results show that due to the presence of hydrogen bonds, many water molecules will be adsorbed around the side chain R, and many hydrogen bonds will break during the stretching process. NH_4_^+^ ions in the training solution protonate the binding site of the hydrogen bond, and the anion SO_4_^2−^ coordinates at the protonated site, which has a reinforcing effect [39]. However, the coordination energy of SO_4_^2−^ at certain sites is much lower than that of H_2_O, thus it cannot play a reinforcing role. As shown in Fig. 3h, when the calculated binding energy between the amino acid fragment and water molecules is between the protonation energy (binding energy in GelMA and NH_4_^+^) and the strengthening energy (binding energy in GelMA and SO4^2−^), the amino acid will produce a reinforcing effect. At this point, it can be found through calculation that the GLU, LEU, and ARG amino acids (the content is less than 15% [33]) do not produce a reinforcing effect, while the remaining amino acids play a significant enhancing role in intensive training. SO_4_^2−^ will not replace the H_2_O bound to the three amino acids^−^ coordination, and the remaining amino acid molecular chains are strengthened by salt ion coordination.

During the stretching training process, since the binding energy is lower than the strengthening binding energy of the coordination, H_2_O is strengthened by SO_4_^2−^ coordination. As the stretching process continues, the molecular chains become closer and oriented along the stretching direction due to the action of tensile stress. When the coordination energy of SO_4_^2−^ is higher than that of water molecules, the water molecules originally bound to the molecular chain will be replaced by SO_4_^2−^. Due to the changes in ionic strength and binding energy, the water molecules gradually dissociate from the GelMA molecular chain (Fig. 3i), which increases its density and realizes the strengthening process of the hydrogel scaffold. To conclude, the highly oriented molecular chains and the dense molecular chain network synergistically significantly improve the mechanical properties of the hydrogel scaffold.

Further analysis from the combined state of microscopic particles. H_2_O in the initial hydrogel is hydrogen-bonded to the hydrogel molecular chain, and SO_4_^2−^ in the tough hydrogel is hydrogen-bonded to the molecular chain. Calculate the energy change of two particles in a hydrogen bond as a unit. For the wave function $\varphi \left(r,t\right)$ of a moving particle in the potential field $V\left(r\right)$, it satisfies the Schrödinger equation:

6 $$i\cdot h\cdot \frac{\partial }{\vartheta t}\cdot \varphi \left(r,t\right)=\left[-\frac{{h}^{2}}{2\cdot m}\cdot {\nabla }^{2}+V\left(r\right)\right]+\varphi \left(r,t\right)$$

where $i$ is the imaginary unit, $h$ is Planck’s constant, $\varphi \left(r,t\right)$ is the wave function, $\nabla$ is the Hamiltonian operator.

The training solution is ammonium sulfate solution, so the potential field $V\left(r\right)$ does not change over t. At the same time, H_2_O or SO_4_^2−^ and GelMA hydrogel are combined by intermolecular force, and the total energy of the single particle is:

7 $$H=-\frac{{\text{h}}^{2}}{2\cdot {m}_{e}}\cdot \sum_{i}{\nabla }^{2}-\sum_{i}\sum_{j}\frac{Z\cdot {e}^{2}}{\left|{R}_{I}-{r}_{i}\right|}+\frac{1}{2}\cdot \sum_{ij\left(i\ne j\right)}\frac{{e}^{2}}{\left|{r}_{i}-{r}_{j}\right|}$$

where $Z$ is the number of electrons of the O atom hydrogen-bonded to the H atom of the scaffold molecular chain, $i$ is the number of electrons of the H atom, and $j$ is the number of electrons of the atom hydrogen-bonded to it, ${R}_{I}$ is atomic radius, ${r}_{i}$ is the distance between electron and nucleus.

For the H atom, there is only one extranuclear electron. Taking this as the origin, the Schrödinger equation is further optimized (Fig. 3j).

8 $$H=-\frac{{h}^{2}}{2\cdot {m}_{e}}\cdot \sum_{i}{{a}_{1}}^{2}-\sum_{I=1}\frac{Z\cdot {e}^{2}}{\left|{R}_{I}-{r}_{i}\right|}+\frac{1}{2}\cdot \sum_{ij\left(i\ne j\right)}\frac{{e}^{2}}{\left|{r}_{i}-{r}_{j}\right|}$$

where ${a}_{1}$ is the distance between the electron and the central proton, ${m}_{e}$ is the rest mass of the electron, and $e$ is the charge carried by a single electron.

1. Before training, the hydrogen bonding energy of the hydrogel scaffold is mainly provided by H_2_O, so Eq. (8) is simplified to:

9 $${H}_{1}=-\frac{{h}^{2}}{2\cdot {m}_{e}}\cdot {{a}_{1}}^{2}-\frac{8{e}^{2}}{{a}_{1}}+\frac{1}{2}\cdot \sum_{j}\frac{{e}^{2}}{{a}_{1}+\Delta i}$$

where $\Delta i$ is the distance between hydrogen atom electrons. h = 6.63 × 10^–34^ J·s, m_e_ = 9.11 × 10^–31^ kg, e = 1.6 × 10^–19^,$($ *j* = 1, 2, 3, 4, 5, 6, 7, 8 and $\Delta \approx 0)$, so

10 $${H}_{1}=-\left(22.18\cdot {{a}_{1}}^{2}+10.24\cdot \frac{1}{{a}_{1}}\right)\times {10}^{-38}$$

2. After training

After training, the hydrogel scaffold plays a coordination role due to SO_4_^2−^. A hydrogen bond-strengthened coordination model is established based on Gillespie's hypervalent structure of sulfate [38–40]. Therefore, SO_4_^2−^ replaces water after training. A schematic diagram of the hydrogen bonding between the molecule and the scaffold molecular chain is shown in Fig. 3k. Among them, after SO_4_^2−^ coordination, 1/2 of the hydrogen bonds are provided by the 8-electron O atoms in the SO_4_^2−^. hypervalent structure, and the 9-electron O atoms offer 1/2 of the hydrogen bonds. Therefore, the overall unit energy after strengthening can be calculated by formula (11)

11 $${H}_{s}=\left[\frac{1}{2}\cdot H+\frac{1}{2}\cdot \frac{9}{8}\cdot H\right]\times \frac{{V}_{{H}_{2}O}}{{V}_{{SO}_{4}^{2-}}}\times \frac{{E}_{{SO}_{4}^{2-}}}{{E}_{{H}_{2}O}}\times \frac{{D}_{{SO}_{4}^{2-}}}{{D}_{{H}_{2}O}}+{H}_{2}$$

where ${H}_{s}$ is the total energy of the particle unit after strengthening, ${V}_{{SO}_{4}^{2-}}$ is the volume of space occupied by SO_4_^2−^ when hydrogen bonded (which is 19,717.312 Å^3^), and ${V}_{{H}_{2}O}$ is the hydrogen is the volume of space occupied by H_2_O when hydrogen bonded (which is 339,725.317 Å^3^) (Fig. 3l). ${E}_{{SO}_{4}^{2-}}$ is the charge carried by the SO_4_^2−^ (11.8), ${E}_{{H}_{2}O}$ is the charge carried by the H_2_O (4.4) (Fig. S4). ${D}_{{SO}_{4}^{2-}}$ is the SAXS test of the tough hydrogel scaffold, the integral of the two-dimension azimuth in the tensile direction (4862.85). ${D}_{{H}_{2}O}$¼ is the SAXS test of the initial hydrogel scaffold, the integral of the two-dimension azimuth of all direction (679.20) (Figs. 2b and S5). ${H}_{2}$ is the total unit energy of particles after training when the water molecule is salted out and stretched away from the molecular chain of the scaffold (Calculated by formula 10).

Equation (11) is calculated as:

12 $${H}_{s}=-\left(7796.38\cdot {{a}_{2}}^{2}+3599.41\cdot \frac{1}{{a}_{2}}\right)\times {10}^{-38}+{H}_{2}$$

The unit energy before and after training is shown in Fig. 3m. Calculated previously ${a}_{1}=2.42$, ${a}_{2}=3.48$, So ${H}_{1}=-134.12\times {10}^{-38},{H}_{s}=-\text{95,723.14}\times {10}^{-38}$. It is calculated that the strength of the scaffold increases by approximately 713 times after training, which is higher than the actual measurement multiple (622). We speculate that this is due to minor structural defects produced during the actual training process.

X-ray diffraction (XRD) was used to characterize the crystalline domains of the PTC and PCT scaffold. As shown in Fig. 3n, after training, there were obvious crystalline aggregates in the PTC and PCT scaffold. The initial scaffold has no apparent crystalline peaks; therefore, it was translucent. This also verified that the orientation and crystallinity of the hydrogel scaffold increased through training, thereby enhancing the mechanical properties.

In the training process, SO_4_^2−^ and NH_4_^+^ enter the hydrogel scaffold from the solution and coordinate to replace the water molecules. Water molecules with binding energy between protonation energy and strengthening energy enter the solution, which increases the density of the hydrogel molecular chain and exposes hydrophilic amino acids. At the same time, due to the presence of stretching, the hydrogel molecular chains become rearranged and highly ordered along the stretching direction, and the mechanical properties are improved. As shown in Fig. 3o, p, the charge distribution of the 13 amino acid fragments was calculated. After training, the water molecules move away from the molecular chain and finally complete the strengthening process.

It is worth noting that ammonium sulfate only acts as a strengthening agent and cross-linking agent during training and can be washed with PBS solution after training. After completing the coordination and directional arrangement of the auxiliary molecular chains, the hydrogel undergoes a 405-nm ultraviolet light cross-linking chain reaction to form a polymer molecular network finally. The trained molecular chain network can be fixed to maintain the high-strength properties of the hydrogel scaffold.

### Printability of the GelMA Ink

**Fig. 4:**
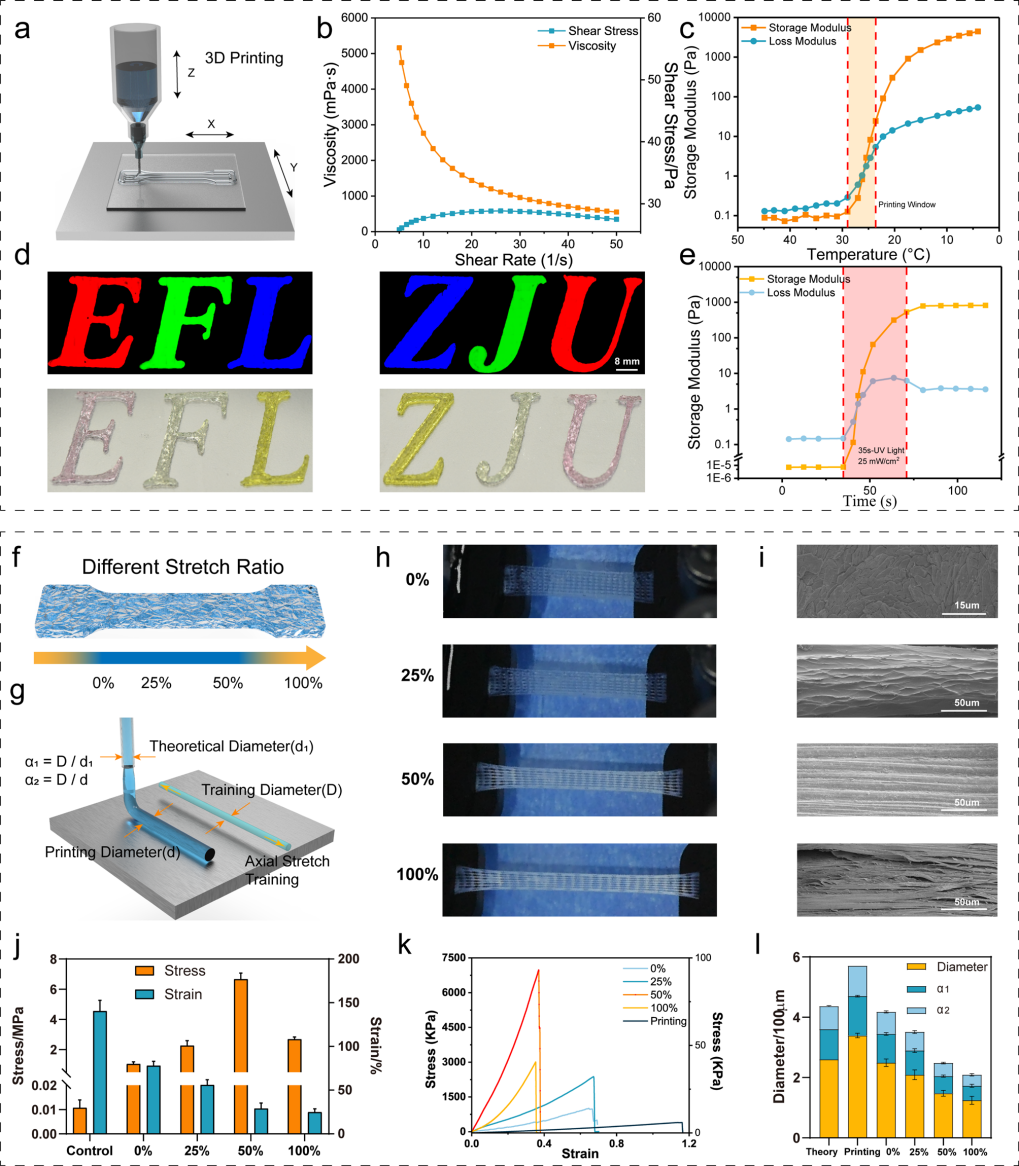
Printability and influence of stretch ratio of tough hydrogel scaffolds. **a** Schematic diagram of 3D printing. **b** Shear thinning properties. **c** Temperature-sensitive properties. **d** “EFL” and “ZJU” printing. scale bar = 8 mm **e** Photo-cross-linking properties. **f** Schematic diagram of stretch ratio. **g** Schematic diagram of scaffold fiber through training processing. **h** Photos of tough hydrogels with different stretching ratios. **i** SEM images of tough hydrogels with different stretch ratios. **j** Stress–strain histogram of tough hydrogels in the different stretch ratio. **k** Stress–strain curves of tough hydrogels in the different stretch ratio. **l** Improved resolution of tough hydrogels

The reversible cross-linking process of GelMA hydrogel was achieved by low temperature. Because GelMA hydrogel needs to undergo many processes, including bio-3D printing (Fig. 4a), saline solution stretching training, PBS buffer release and UV cross-linking, for GelMA, it is necessary to maintain the original gelation state until the GelMA precursor solution is fully photo-cross-linked. Additionally, the GelMA precursor solution should be in a semi-gelation condition during the extruding process to guarantee printability and the production of stable and uniform filaments.

In terms of the 3D printing process, the flow step measurement of GelMA ink was carried out to explore its printability. By testing the viscosity of the hydrogel at different shear speeds, we verified that as the shear rate increases, the viscosity of the GelMA hydrogel solution gradually decreases, which means that it has shear thinning properties and is suitable for extrusion bioprinting (Fig. 4b). By testing the changes in storage modulus and dissipation modulus of the hydrogel solution from 5 to 45 °C, it was verified to be in a semi-gel state at 24–28 °C (printing window) (Fig. 4c). It was finally confirmed that the hydrogel could complete the transition from solution state to gel state under a UV light source of 25 mW cm^−2^ (Fig. 4d), and the mechanical stretching and salting-out-assisted strengthening process of the tough hydrogel could be accomplished. Curing is completed through 405 nm UV light, maintaining its ordered molecular chain structure generated through training to enhance the tensile strength. Figure 4e shows the "EFL" and "ZJU" characters printed by bio-extrusion, which verifies the printability of GelMA hydrogel. The upper picture was taken using a fluorescence confocal microscope, and the lower picture is a photograph of the actual object.

### Effect of Mechanical Stretching on Strengthening Training Processing

During the mechanical stretching and salting-out-assisted training process of hydrogel, different stretching ratios will affect the compactness of the molecular chains. At the beginning of stretching, as the stretching ratio increases, the effect of the arrangement of molecular chains along the stretching direction becomes more obvious, and the strengthening effect is also more pronounced. However, if the tensile ratio is too large, the hydrogel fiber may be broken, resulting in microstructure defects and then affecting the mechanical properties of the hydrogel scaffold.

To verify the synergistic strengthening effect of stretching and saline solution, we studied the presence of stretching (the ratio is 0%) and different stretching ratios (25%, 50%, and 100%) on the strengthening effect. Surprisingly, although the Hofmeister effect has a salting-out effect on the protein, which can enhance the properties of the hydrogel [26], mechanical properties of the strengthened hydrogel obtained when the stretching ratio is 0% (soaked in (NH_4_)_2_SO_4_ solution) are significantly smaller than other groups.

As shown in Fig. 4f, the hydrogel has different morphologies under four different stretching ratios of 0%, 25%, 50%, and 100%. The initial appearance of the hydrogel is transparent and colorless. As the stretching ratio increases, the color of the hydrogel gradually tends to become white, which means denser fibers (Fig. 4g, h). More importantly, due to the higher degree of orientation of the molecular chains in the fibers and the denser fiber arrangement, the tensile strength of the tough hydrogel scaffold gradually increases as the stretching ratio rises (0–50%). However, considering that the fibers are trained from disordered to a parallel arrangement state, during further stretching, some fibers may be damaged by excessive tensile force and deformation. Broken fibers in turn cause defects inside the hydrogel scaffold, affecting the mechanical properties (Fig. 4i-k). Besides, compared with the initial hydrogel, the stretching training process makes the fiber diameter of the hydrogel scaffold smaller, which also effectively improves the printing resolution (Fig. 4l).

### Mechanical Properties of the Tough Hydrogel Scaffolds

**Fig. 5:**
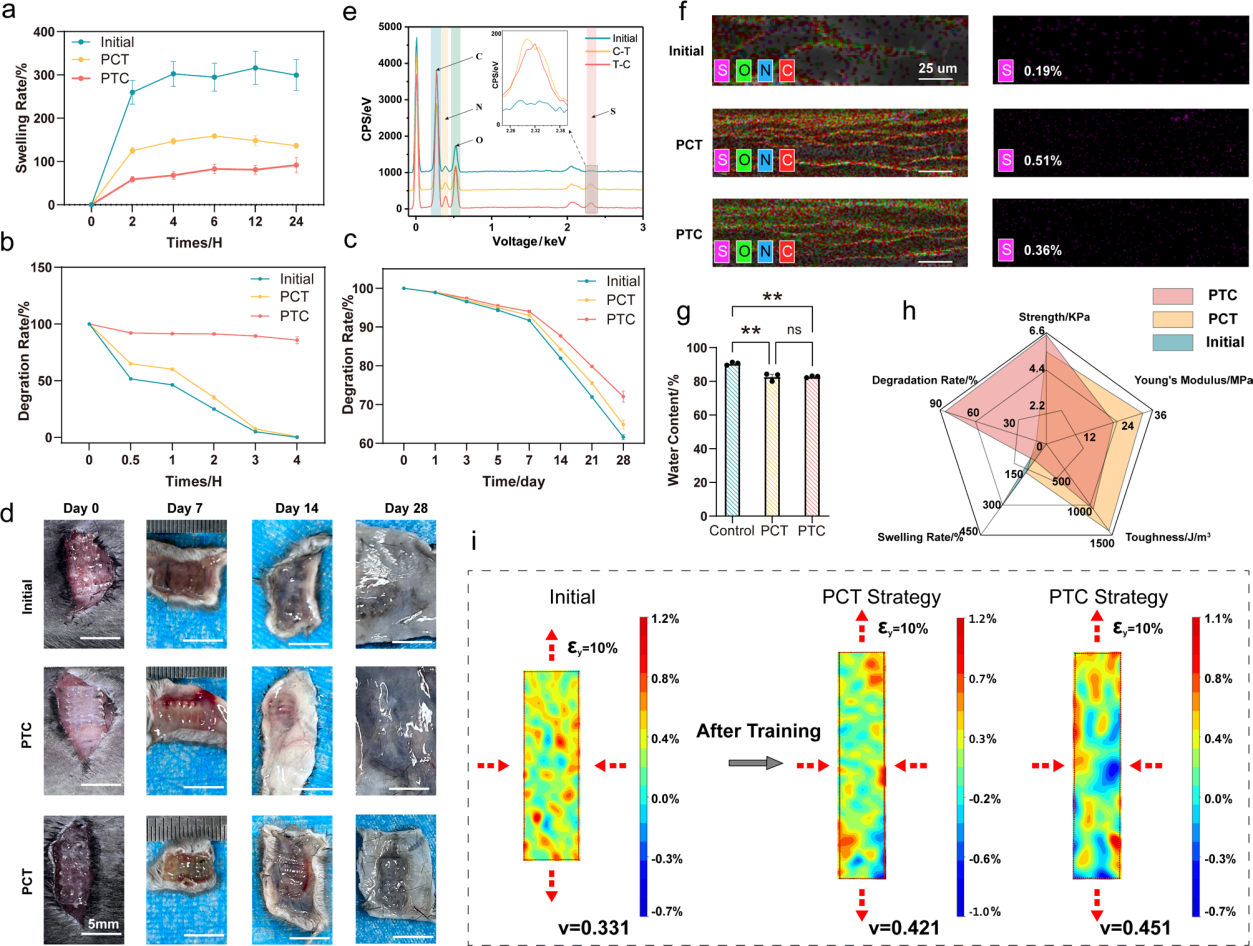
Mechanical properties of tough hydrogels. **a** Swelling rate. **b** Degradation rate in *vitro* in type II collagenase. **c** Degradation rate in *vitro* in PBS. **d** Degradation rate in *vivo*. scale bar = 5 mm **e** Elemental analysis. **f** EDS analysis of Initial, PCT and PTC hydrogel scaffold. scale bar = 25 μm **g** Water content **h** Radar chart **i** Poisson's ratio of initial, PTC, and PCT tough hydrogels scaffolds

Mechanistically, directional stretch training forms aligned structures in the GelMA hydrogel [18], whereas salting out increases the local concentration of the GelMA hydrogel to values above the nominal concentration and strongly induces the aggregation and crystallization of GelMA hydrogel by phase separation to form nanofibrils [20]. Due to the increase in hydrogel fiber aggregation and local concentration, the swelling properties of the trained hydrogels are significantly improved compared to the initial hydrogels. As shown in Fig. 5a, the swelling rate of hydrogel strengthened by the PCT strategy is 148.571% after 12 h and only 81.134% after the PTC strategy. A low swelling rate is crucial for maintaining the overall structure of hydrogel scaffolds, which contributes to the effect of tissue repair in vivo. More importantly, the highly aligned fiber arrangement and tight hydrogel structure also have a significant effect on delaying degradation. After adding collagenase II solution, the tough hydrogel prepared by the PCT strategy degraded to 92.71% after 3 h, whereas the tough hydrogel prepared by the PTC strategy only degraded to 10.66% after 3 h. The initial hydrogel is left with less than 5% of the initial mass (Fig. 5b). The degradation experiment in PBS also verified the above conclusion. The tough hydrogel scaffold trained by PTC can effectively delay the degradation rate (Fig. 5c). We further proved that the hydrogel after training could effectively delay the degradation rate through the degradation experiment in *vivo* (Fig. 5d).

During intensive training in a saline solution, salt ions will enter the hydrogel to assist in enhancing the orientation effect of stretching on the molecular chain. Due to different binding energies, water molecules are snatched away to increase the tightness of the molecular chain network, while salt ions serve as auxiliary hydrogel interactions. The cross-linking agent should be washed with PBS solution after completing the training. Importantly, the ions serve only as gelation triggers and property modulators. They are not necessary to remain in the gel, maintaining the high biocompatibility of GelMA without excess ions [40]. As an auxiliary strengthening cross-linking agent, ammonium sulfate can be cleaned away by PBS after training and does not exist in the tough hydrogel [27]. As shown in Fig. 5e, f, the sulfur contents of the tough hydrogels prepared by the PCT and the PTC strategies were 0.51% and 0.36%, respectively, a slight increase compared to the 0.19% of the initial hydrogel, but neither affected their biocompatibility. The water content of human muscle tissue is about 70%-80% [18] and that of untrained hydrogel is about 90.58%. The water contents of hydrogels prepared by the PCT and PTC strategies were about 82.79%, higher than that of human muscle tissue (Fig. 5g). The performance differences between the tough hydrogel scaffolds prepared by the PCT strategy and the PTC strategy and the initial hydrogel are shown in Fig. 5h. The former two are significantly superior to the initial hydrogel in tensile strength, Young's modulus, toughness, swelling, and degradation.

The movement of the human body depends on the skeletal muscles, which are attached to the bones [41], which are the most abundant tissue in the human body accounting for 40%–50% of the body mass. The Poisson's normal human soft tissue ratio is about 0.45–0.5, 0.493 for the relaxed muscles and 0.480 for contracted muscles [42]. The Poisson's ratio of the tough hydrogel scaffold prepared by the PCT strategy was 0.421 and that of the hydrogel prepared by the PTC strategy was 0.451. Compared with the initial hydrogel (0.331), the Poisson's ratio of the two is closer to that of human tissue (Fig. 5i). We speculate that this is because when the hydrogel is pre-stretched during the training-enhanced hydrogel process, it stretches the molecular chains in the stretching direction (longitudinal) and has more parallel alignment, while stretching that is perpendicular to the hydrogel in the training direction (transverse direction) maintains the structure prepared by 3D printing. Therefore, when the tensile test is repeated, a smaller longitudinal tensile strain will have a larger transverse strain, so the Poisson's ratio will be larger.

### Broad-Range Tunable Mechanical Properties of the Tough Hydrogel Scaffold

**Fig. 6:**
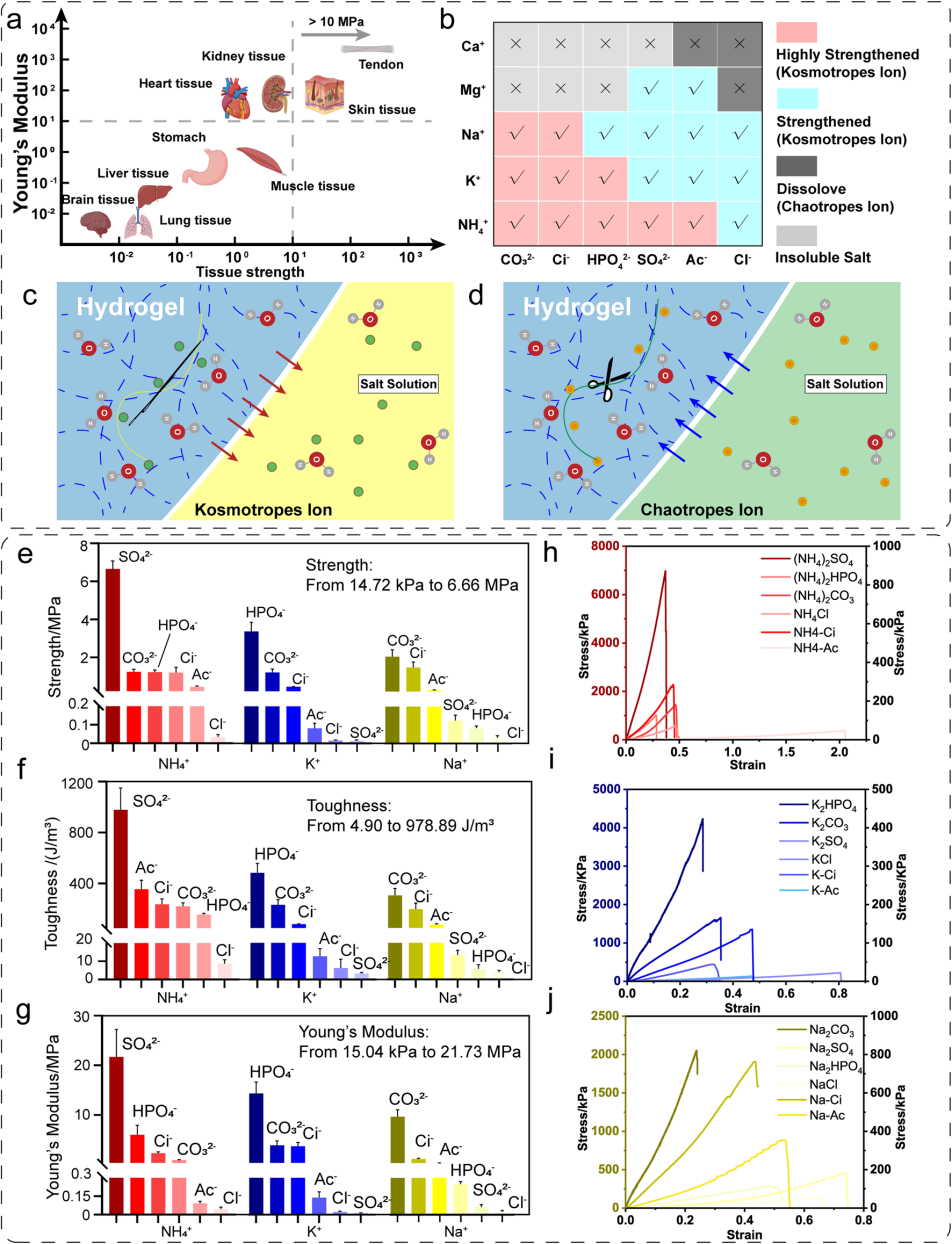
Adjustable mechanical properties of tough hydrogel. **a** Mechanical property of human tissue. **b** Salt ion strengthening effect. **c** Schematic diagram of kosmotrope salts strengthening. **d** Schematic diagram of chaotrope salts strengthening. **e** Tough hydrogel strength under various salt conditions. **f** Tough hydrogel toughness under various salt conditions. **g** Tough hydrogel Young’s modulus under various salt conditions. **h** Stress–strain diagram of tough hydrogels trained using ammonium salts. **i** Stress–strain diagram of tough hydrogels trained using potassium salts. **j** Stress–strain diagram of tough hydrogels trained using sodium salt

The severity of mechanical mismatch may damage the tissues. Furthermore, the elastomer's non-degradability makes it unsuitable for implantation [43]. GelMA is a 3D-cross-linked material with a composition like human tissue. We prepared a tough hydrogel scaffold by proposing a method of mechanical training and salting-out-assisted photo-cross-linking, which can be applied in customized tissue engineering repair. More importantly, by adjusting the type of salt ions used in mechanical training, the mechanical properties of the tough hydrogel can be further conditioned to match the mechanical properties of different human tissues [42, 44] (Fig. 6a).

An order of ions known as the Hofmeister series has the power to change the solubility of proteins. The Hofmeister effect has received extensive research since Hofmeister first introduced the series in 1888. The following is the typical order of anions: CO_3_^2−^ > SO_4_^2−^ > S_2_O_3_^2−^ > H_2_PO_4_^−^ > F^−^ > CH_3_COO^−^ > Cl^−^ > Br^−^ > NO_3_^−^ > I^−^ > ClO_4_^−^ > SCN^−^ [28]. Anions have been shown to have a more noticeable influence than cations [45]. Kosmotropic (well-hydrated) ions are on the left side of the series, and chaotropic (poorly hydrated) ions are on the right. The former decreases, and the latter increases protein solubility. In the previous strengthening mechanism analysis section, we discussed the strengthening mechanism of ammonium sulfate on GelMA hydrogel. Herein, we further studied the performance-enhancing effects of different ions in the Hofmeister effect ion sequence on GelMA hydrogels. The effect of kosmotropes ions on GelMA hydrogel is shown in Fig. 6c. The hydrogen bonds between the hydrogel and its hydration water molecules are firstly made unstable by the ability of some anions to polarize the water molecules. The salt ions form hydrogen bonds with the water molecules in the hydrogel, gradually forming new hydrogen bonds between the peptide chains in the hydrogel. The ions shorten the distance between the hydrogel molecular chains due to the salting-out effect like needle threads and then enhance the hydrogel mechanical properties (Fig. 6c). As for chaotropic ions, they are the salts that disrupt the hydrogen bonds between water molecules and increase their disorder. Chaotropic ions efficiently salt proteins in the solution, destroying their 3D configuration and decreasing the solutions’ surface tension and viscosity [46]. The ions act like scissors, disrupting the connections between the peptide chains of GelMA, increasing its solubility in water and ultimately reducing its mechanical properties, potentially even causing it to dissolve in salt solutions (Fig. 6d).

According to the Hofmeister series, a series of sodium salts and chloride salts were chosen to methodically evaluate the impact of each type of anion/cation. The strengthening effect of each salt on the GelMA hydrogel scaffold during training was analyzed separately (Fig. 6b). We evaluated the strengthening effects of ammonium ions, potassium ions, sodium ions, magnesium ions and calcium ions on hydrogels during mechanical stretching training. The typical stress–strain curves of GelMA hydrogels treated with various ammonium salts selected based on the Hofmeister series are shown in Fig. 6e. The GelMA hydrogel trained in (NH_4_)_2_SO_4_ solution has the highest ultimate stress (6.66 MPa), toughness (978.89 kJ m^−3^) and Young’s modulus (21.73 MPa) among the anion series, whereas the GelMA hydrogel trained in NH_4_Cl has the lowest ultimate stress (34.48 kPa) (Fig. 6f–h). In ammonium salt, the GelMA hydrogels of different anions were systematically ranked in the order of mechanical properties as follows: SO_4_^2−^ > HPO_4_^2−^ > CO_3_^2−^ ≈ Ci > Ac^−^ > Cl^−^. The typical stress–strain curves of GelMA hydrogels treated with various potassium salts are shown in Fig. 6i. The mechanical properties of tough GelMA hydrogel scaffolds mechanically trained in potassium salts are lower than those trained in ammonium salts, which exhibit the highest ultimate stress (3.36 MPa), toughness (470.89 kJ m^−3^) and Young’s modulus (14.38 MPa) among the anion series, whereas the GelMA hydrogel trained in K_2_SO_4_ has the lowest ultimate stress (14.72 kPa), toughness (4.9 kJ m^−3^) and Young’s modulus (15.04 kPa) (Fig. 6f–h). In potassium salt, the GelMA hydrogels of different anions were systematically ranked in the order of mechanical properties as follows: HPO_4_^2−^ > CO_3_^2−^ > Ci > Ac^−^ > Cl^−^ ≈SO_4_^2−^. The typical stress–strain curves of GelMA hydrogels treated with various sodium salts are shown in Fig. 6j. Sodium-trained hydrogels have the lowest mechanical properties among the three cations, which exhibit the highest ultimate stress (2.05 MPa), toughness (306.65 kJ m^−3^) and Young’s modulus (9.72 MPa) among the anion series, whereas the GelMA hydrogel trained in NaCl has the lowest ultimate stress (32.02 kPa), toughness (9.93 kJ m^−3^) and Young’s modulus (25 kPa) (Fig. 6f–h). In sodium salt, the GelMA hydrogels of different anions were systematically ranked in the order of mechanical properties as follows: CO_3_^2−^ > Ci > Ac^−^ > SO_4_^2−^ > HPO_4_^2−^ > Cl^−^.

The anion sequences corresponding to the above ammonium ions, potassium and sodium ions belong to kosmotropic salts. During the stretching training process, as the stretching ratio increases, the fiber grid is aligned, the distance between the nanofibrils is significantly reduced, and salting out occurs at the same time, which increases the density of molecular chains. During the salting-out process, abundant hydrogen bonds are formed between hydrogels, and the GelMA hydrogel scaffold is strongly aggregated and partially crystallized, which comprise opaque crystalline aggregates that cause light to scatter, giving the hydrogel its white look (Fig. S7). Magnesium salts and calcium salts are chaotropic salts. During intensive training of the components of the saline solution, such as salts, the hydrogel will soften or dissolve to varying degrees. During the training process, the chaotropic salt did not cause the GelMA hydrogel scaffold to produce opaque white crystalline areas, and the hydrogel remained transparent and colorless (Fig. S7). More importantly, when strong chaotropic anions were combined with chaotropic cations, the GelMA hydrogel scaffold quickly dissolved during training and could not maintain its 3D-printed structure.

In summary, by changing the types of kosmotropic and chaotropic salts during mechanical training and salting-out assisted intensive training, GelMA hydrogel scaffolds with different mechanical properties can be obtained, which means that they can better match the mechanical properties of various soft and hard tissues of the human body.

### Broad-Range Hydrogel Applicability of the Tough Hydrogel Scaffold

It has been verified that the proposed PTC and PCT intensive training methods are suitable for gelatin hydrogels and can adjust the mechanical properties to match various soft and hard tissues of the human body. Here, we further demonstrate that this is a versatile method that is applicable to many types of hydrogels.

As discussed above, the Hofmeister effect, also known as the ion-specific effect, is the property of different salts that allows them to precipitate proteins from aqueous solutions distinguishably [47]. Mechanical stretching and cycling training can also position the hydrogel molecular chain to achieve enhanced properties; the hydrogel fibers are oriented along the drawing direction, and consequently the orientation of GelMA crystallites and polymer chains change along the stretching direction [22]. To explore the strengthening effects of mechanical stretching and salting out on different hydrogels, we selected various types of hydrogel scaffolds for strengthening training.

**Fig. 7:**
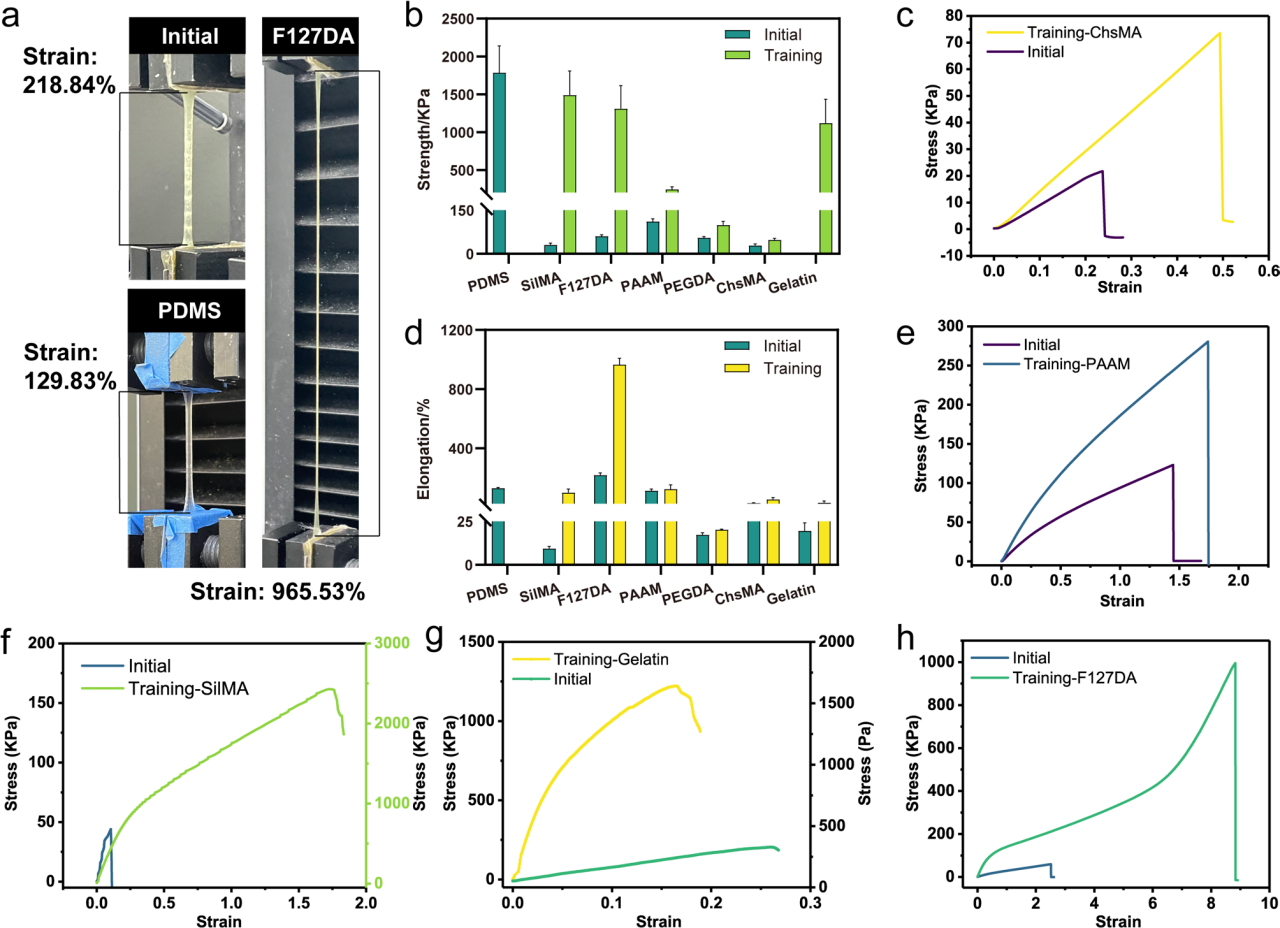
Universal method for tough hydrogel scaffolds. **a** Comparison of strain photos between reinforced F127DA and PDMS. **b** Comparison of strength before and after training with different types of hydrogels. **c** Stress–strain diagram before and after ChsMA training. **d** Comparison of strain before and after training with different types of hydrogels. **e** Stress–strain diagram before and after PAAM training. **f** Stress–strain diagram before and after SilMA training. **g** Stress–strain diagram before and after gelatin training. **h** Stress–strain diagram before and after F127DA training

The most obvious strengthening effect was obtained with protein hydrogel scaffolds, such as silk fibroin hydrogel and gelatin. The tensile strength of silk fibroin hydrogel (SilMA) after intensive training was 1487.64 kPa, which is 48.57 times (30.63 kPa) that of the initial hydrogel (Fig. 7b, f). After intense training, the tensile strength of gelatin was 1117.34 kPa, which is 2794.81 times (0.4 kPa) greater than the initial strength of the hydrogel (Fig. 7b, g). Meanwhile, this method of preparing tough hydrogels also has a significant reinforcing effect on the polymer hydrogel, such as polyether F127 diacrylate(F127DA), polyacrylamide (PAAM), and polyethylene glycol diacrylate (PEGDA) (Fig. 7b-h). Surprisingly, this strengthening method has a significant toughness enhancement effect on F127DA (Fig. 7d). As shown in Fig. 7a, the breaking strain of untrained F127DA is 218.84%, and that of trained F127DA is enhanced to 965.53%. The latter has extremely high elasticity and can be stretched to about 10 times its original length, which is significantly higher than the ductility of PDMS. We speculate that F127DA is a typical non-ionic amphiphilic polymer consisting of hydrophilic PEO and hydrophobic PPO groups. An aqueous solution can self-assemble into micelles with PPO as the core and PEO as the shell [48]. Therefore, untrained F127DA has high toughness. After training, the self-assembled micelles are further rearranged, and the anisotropy is enhanced to have extremely high toughness, especially in the elongation at break. Stretchability plays a crucial role in various fields, particularly in the area of strain sensors [49]. For polysaccharide hydrogels, the preparation method of our proposed tough hydrogel scaffolds also has a certain strengthening effect, such as chondroitin sulfate hydrogel (ChsMA) and hyaluronic acid hydrogel (HAMA) (Figs. 7b–d and S8).

### Biological Application of Tough Hydrogel Scaffold

Gelatin methacryloyl (GelMA), a synthesized biomacromolecule, exhibits excellent biocompatibility and formability. Its main component, gelatin, has biological properties resembling the extracellular matrix (ECM) [14]. For biomaterials to be used in clinical settings, good cytocompatibility is essential.

**Fig. 8:**
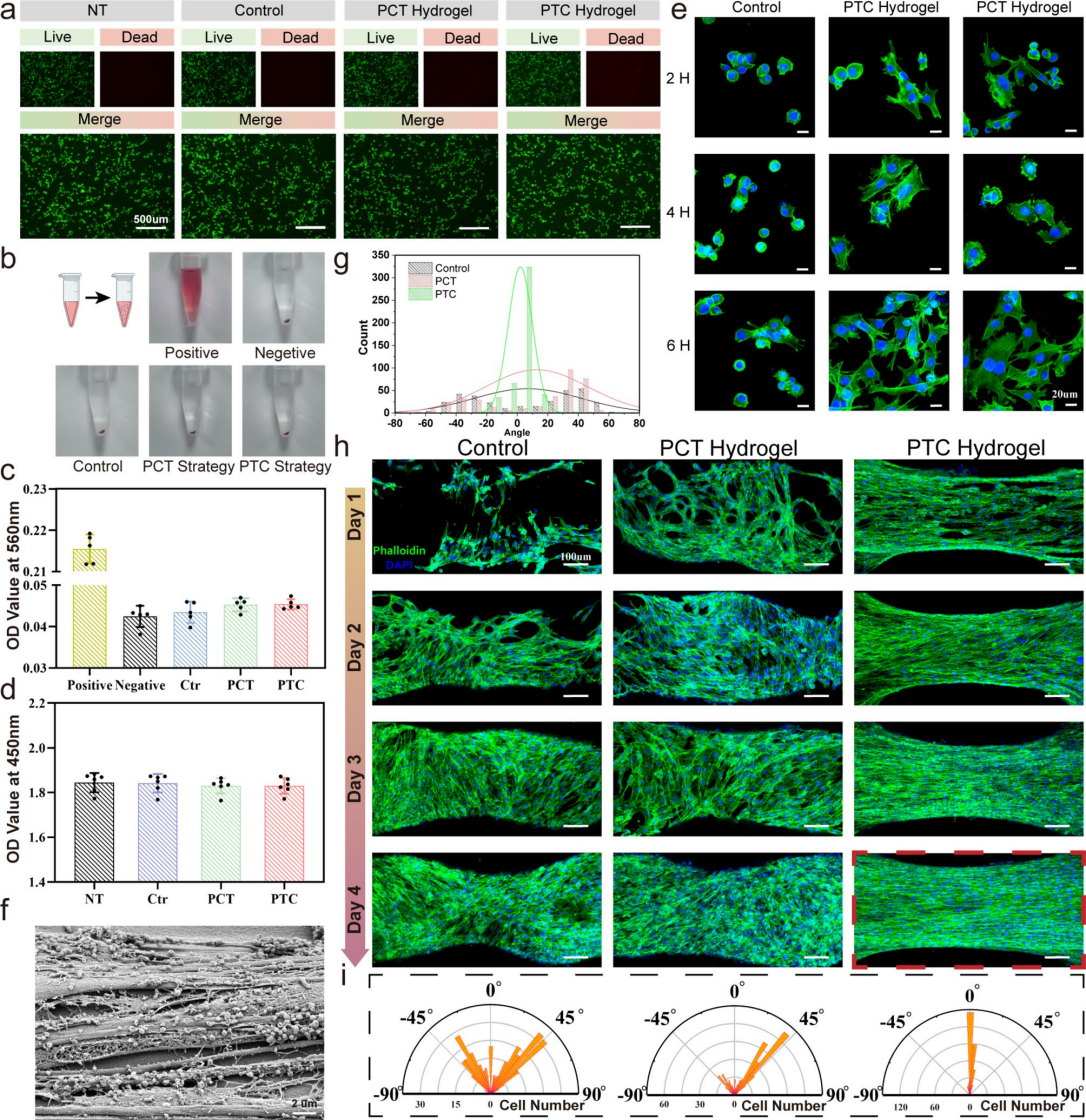
Biocompatibility comparison between the initial hydrogel and PTC & PCT tough hydrogel. **a** Live-Dead staining after C2C12 cultured for 24 h, scale bar = 500 μm. **b** Hemolysis test of hydrogels. **c** Hemolysis rate of hydrogels. Data is presented as mean ± SD, *n* = 5, ***p < 0.001 compared to the Positive group. **d** CCK-8 assay after C2C12 cultured for 24 h. **e** Immunofluorescence stain of β-actin (green) and nuclei (blue) in C2C12 on 2, 4, and 6 h after seeding on hydrogels. Original magnification is 40 × , scale bar = 20 μm. **f** SEM picture of oriented cells, scale bar = 2 μm. **g** C2C12 orientation angle of control, PCT and PTC scaffold. **h** Comparison of cell orientation effects. Original magnification is 10 × , scale bar = 100 μm. **i** Nightingale rose plots of cellular orientation angle

The biocompatibility of the tough hydrogel scaffold was evaluated by live/dead staining assay, and the results are shown in Fig. 8a. Hemolysis test is an important indicator for evaluating the adverse effects of biomaterials on red blood cells, platelets, coagulation, and thrombosis. After being applied to a human body, biomaterials will unavoidably come into contacting with blood, hence it is important to assess the blood compatibility of toughened hydrogel scaffolds. As shown in Fig. 8b, c, compared with the negative control group, all group showed a lack of significant statistical differences in hemolysis and hemolysis. Further, through the CCK-8 experiment, we verified that the tough hydrogel scaffold, after intensive training, still has excellent biocompatibility Fig. 8d. The above results indicate that the toughened hydrogel scaffold has good biocompatibility and hemocompatibility, laying the foundation for further animal experiments in vivo.

In the tissue engineering field, the capacity to adhere to scaffold materials is critical for cellular viability and functionality [50]. Therefore, the surface adhesion characteristics of PTC and PCT tough hydrogels were determined (Fig. 8e). Compared with the initial hydrogel, cells seeded in PTC and PCT hydrogels have a larger spreading area after 2 h later, which indicates that these two hydrogels have better adhesion effects. At 6 h, the initial hydrogel began to spread, while many cells of PTC hydrogel and PCT hydrogel expanded into fusiform at 2 h. We speculate that this is because after the hydrogel scaffold has been trained, the hydrophilic amino acid peptide chain is exposed to the scaffold surface, increasing its surface hydrophilicity. Gelatin is a cheap form of denatured collagen that may be obtained from several sources, yet it still has natural cell binding motifs like RGD, which is conducive to cell adhesion [51]. Therefore, based on the excellent cell adhesion property of GelMA hydrogel itself, intensive training gives the hydrogel scaffold stronger cell adhesion ability.

In the general hydrogel strengthening method, tensile strength and modulus can be customized. Here, taking muscle tissue as an example, based on confirming its mechanical properties and structure bionics, we verify the effect of PTC hydrogel scaffold on cell orientation in vitro and further check its ability to repair large tissue muscle defects.

From a structural standpoint, skeletal muscles tissue possesses a particular architecture where the multinucleated fibers are densely packed to form parallelly aligned bundles [52]. Notably, such hierarchical architecture can also be observed at the single-cell level in skeletal muscle cells. This highly oriented cellular structure has a major impact on muscle fiber size, function, form and the positioning of the nucleus [53].

Surprisingly, when C2C12 cells were seeded onto PTC hydrogel scaffolds, the cells grew directionally along the stretching direction of mechanical training and arranged into highly oriented structures after 4 days (Fig. 8f–i). This is because the hydrogel scaffold forms the functional surface structure, including nanoscale directional molecular chains and micron-scale directional fiber structures during mechanical training, allowing muscle cells to grow directionally on the scaffold [54], signals from the topographic structure are transmitted to the entire mass of cells through the integration of intracellular and intercellular signaling cascades as well as mechanotransduction at cell–cell junctions and cell-ECM interfaces [55], which leads the entire cell population achieved a high degree of directional growth. The highly directional growth of cells also confirmed that tough hydrogel scaffolds can be further used in muscle tissue engineering [56, 57].

This further confirms that the PTC hydrogel scaffold not only has mechanical property that matches muscle tissue but can also guide the directional growth of muscle cells in vitro, verifying the possibility of PTC hydrogel scaffolds to further complete muscle tissue repair. The initial hydrogel and PCT hydrogel scaffolds also have a certain role in guiding the directional growth of cells due to the 3D-printed structure. However, since the PCT hydrogel scaffold has completed photo-cross-linking before mechanical training, the density of the hydrogel molecular network increases, and mechanical training has a lower effect on the rearrangement of molecular chains and fibers, making the cell orientation effect inferior to that of PTC hydrogel scaffold (Fig. 8h). In addition, cross-linking after mechanical stretching makes the hydrogel scaffold prestressed along the stretching direction, which has also been proven to promote directional cell growth [58]. However, because mechanical training can effectively improve the orientation of hydrogel molecular chains and fibers, both PCT and PTC hydrogel scaffolds have a significant directional guidance for cell growth compared with the initial hydrogel, corresponding to the data of Nightingale Rose plots showed results (Fig. 8i).

### PTC Tough Hydrogel for Volumetric Muscle Loss Reconstruction

Within skeletal muscle, there are two primary structures for force transmission and passive load bearing – the extensively studied muscle fiber, where a majority of the passive properties originate from the giant elastic protein [59], and the poorly understood connective tissue structures that surround these fibers, the muscle extracellular matrix (ECM) [60]. According to the work of previous researchers, prepared by Teja Guda's team, collagen and fibrin can effectively promote muscle regeneration in terms of their mechanical properties. The modulus of collagen is 3.7 ± 1.2 MPa, and the modulus of fibrin is 3.3 ± 1.2 MPa, which is within one order of magnitude with muscle compared to literature reports [61].

In terms of mechanical properties, according to the literature, the tensile strength of muscle is less than that of 10 MPa [18, 42]. While other tissues, such as tendons and ligaments, have ultimate tensile strength ranging from 50 to 150 MPa and an elastic modulus of between 1.0 and 2.0 GPa [42]. The PTC hydrogel has a tensile strength (6.66 MPa) close to that of muscle. Regarding Poisson's ratio, PTC hydrogel is consistent with muscle (4.51 vs 4.8) [42]. At the microstructure level, PTC hydrogel has nanoscale oriented molecular chain and micron-oriented microstructure, consistent with the multi-level directional structure of muscle, and verified that the PTC scaffold can induce directional cell growth in vitro. Therefore, we verified that the PTC scaffold can effectively promote muscle fiber growth and accelerate muscle regeneration in vivo.

**Fig. 9:**
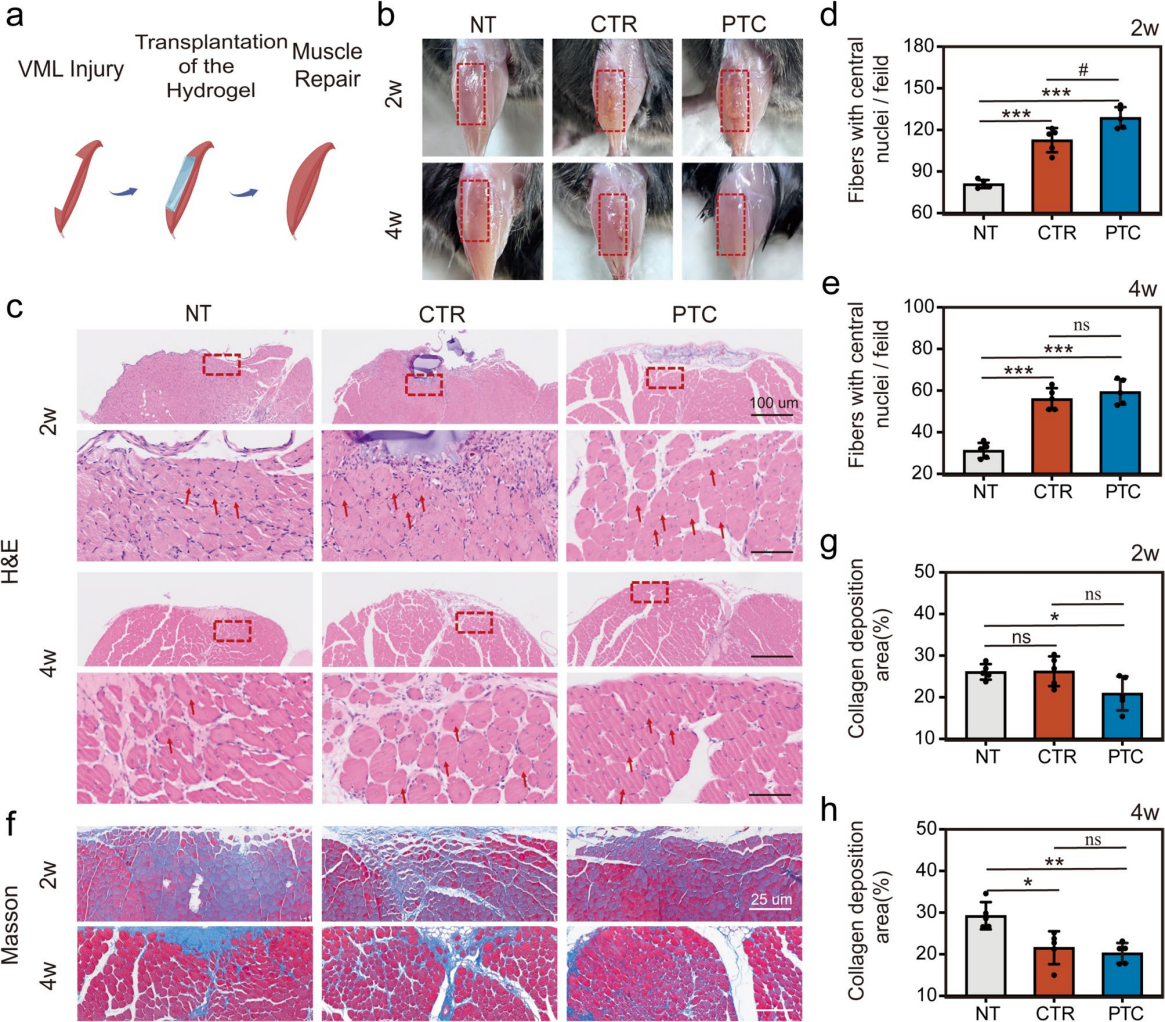
Histological analysis of regenerated muscle in each group at 2-week and 4-week post-surgery. **a** Schematic representation of PTC hydrogel-mediated muscle repair after VML. **b** Representative photographs of the transplanted sites after 2 and 4 weeks. **c** Representative hematoxylin and eosin (H&E) staining images, original magnification is 40 × (scale bar = 500 μm) and 200 × (scale bar = 100 μm), arrows represents fibers with central nuclei. **d** Quantitative analysis of fibers with central nuclei per field at 2 weeks. **e** Quantitative analysis of fibers with central nuclei per field at 4 weeks. **f** Representative masson’s trichrome (MT) stained images, original magnification is 80 × , scale bar = 250 μm. **g** Quantitative analysis of collagen deposition area at 2 weeks. **h** Quantitative analysis of collagen deposition area at 4 weeks. All data are presented as means ± SD, *n* = 4, *p < 0.05, **p < 0.01, ***p < 0.001 compared to NT, #*p* < 0.05 compared to CTR

VML injury, characterized by extensive loss of skeletal muscle tissue, leads to severe functional impairment, including fibrotic tissue deposition, lack of reinnervation, minimal vascular system, and insufficient muscle regeneration to bridge the defect site [62–64]. Recently, various hydrogel materials have been tested for skeletal muscle regeneration [65–67]. We first cut 2 mm×1 mm×7 mm sized blocks of muscle tissue from the anterior tibia muscle of mice to construct VML and then implanted hydrogel scaffold into the defect site. The muscle regeneration was evaluated at 2 and 4 weeks post-surgery in each group (Fig. 9a). We took pictures of the tibialis anterior muscle samples from each group of animals when collecting samples, with some of the mages featuring the overall appearance of each group presented in Fig. 9b. Two weeks following surgery, the muscle defect in the NT group still showed clear defect depression, while more obvious muscle volume recovery with a small amount of hydrogel residue was seen in the CTR and PTC groups. By 4 weeks, the hydrogel of the CTR and PTC groups had entirely broken down, and the muscle tissue surface was smoother and plumper.

Histological examination was conducted to evaluate the early muscle regeneration process in VML. H&E staining can visualize the characteristics of muscle regeneration, such as small-caliber and newly formed tissue, which can be easily observed on the cross-sectional area of the muscle, while the nuclei of mature muscle fibers are located around the periphery of the muscle fibers [68, 69]. The H&E staining results of the NT, CTR, and PTC groups on weeks 2 and 4 are presented in Fig. 9c. Incompletely degraded hydrogels were observed in the defect areas of the CTR and PTC groups after 2 weeks. Four weeks after surgery, a certain amount of immature myofibers with small diameters could be observed in the NT group, and the myofiber diameters were significantly larger in the CTR and TC groups. In addition, the number of newly generated muscle fibers with a central nucleus in the PTC group was significantly increased, which was higher than that in the NT and CTR groups, indicating that the implanted hydrogel can promote muscle regeneration in the early healing process (Fig. 9c–e).

A crucial element in the functional regeneration of skeletal muscles is collagen deposition at the injury site [70]. However, skeletal muscle fibroblasts infiltrate the location of the lesion during the process of producing new muscle fibers and create extracellular viral matrix protein, causing fibrotic scarring. Excessive scarring, in turn, restricts functional recovery and hinders muscle renewal [71]. Thus, we measured the amount of collagen deposition and fibrosis in the cross section of the tibialis anterior muscle by Masson’s trichrome staining (Fig. 9f). In all 3 groups, a modest amount of collagen production was seen at the injury site by 2 weeks post-surgery, which was helpful for the regeneration of muscle fibers. At 4 weeks post-surgery, dense collagen deposition occurred at the injury site in the NT group, leading to the formation of fibrotic tissue. The deposition of collagen fibers was also significant in the CTR group, while this was the lowest in the PTC group (Fig. 9f–h).

**Fig. 10:**
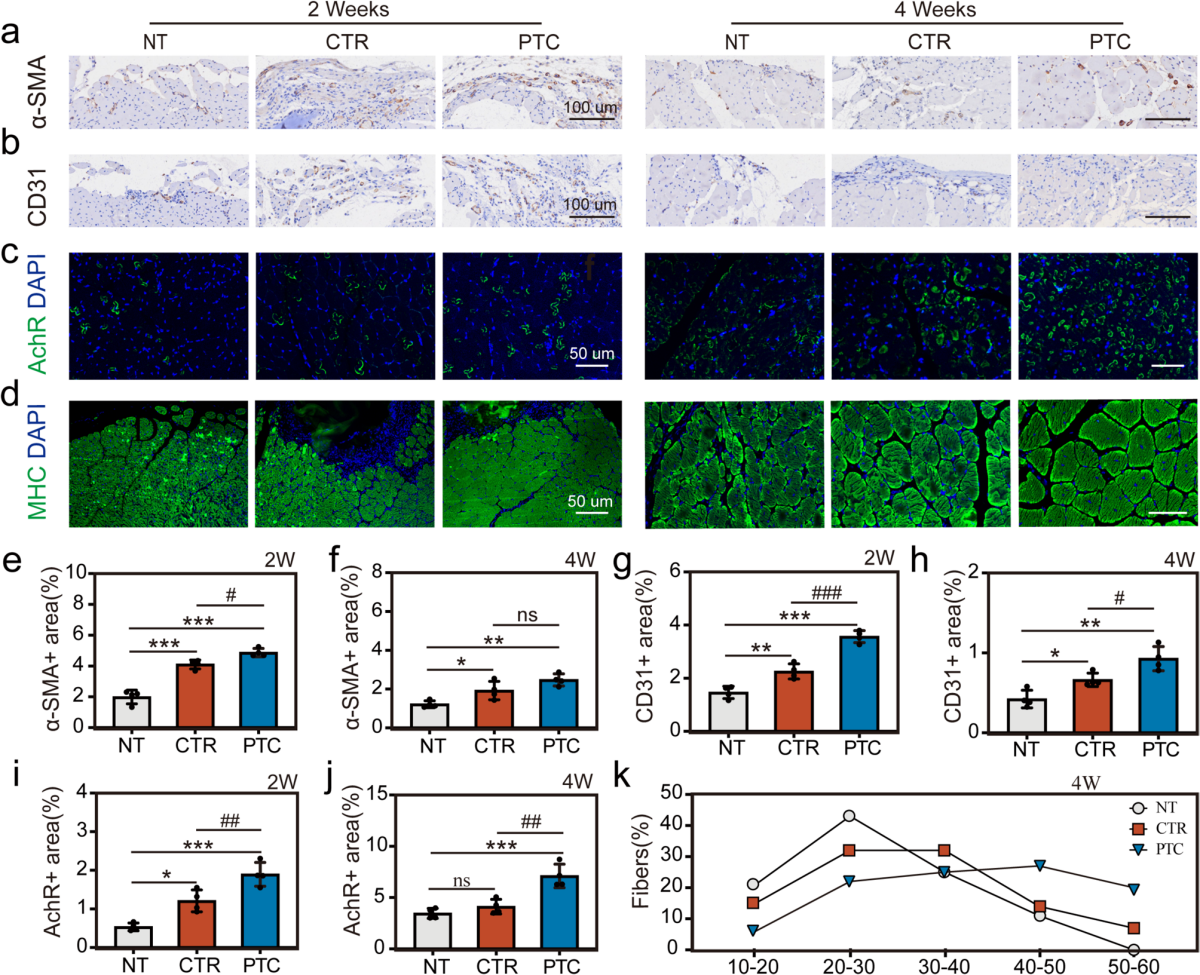
Immunohistochemical analysis of regenerated muscle in each group at 2-week and 4-week post-surgery. **a** Representative immunohistochemical staining images for α-SMA, original magnification is 100 × , scale bar = 100 μm. **b** Representative immunohistochemical staining images for CD31, original magnification is 200 × , scale bar = 100 μm. **c** Representative Immunofluorescent staining images for AchR, original magnification is 400 × , scale bar = 50 μm. **d** Representative immunofluorescent staining images for MHC, original magnification is 200 × (scale bar = 100 μm) and 400 × (scale bar = 50 μm). **e** Quantitative analysis of α-SMA-positive per field at 2 weeks. **f** Quantitative analysis of α-SMA-positive per field at 4 weeks. **g** Quantitative analysis of CD31-positive at 2 weeks. **h** Quantitative analysis of CD31-positive at 4 weeks. **i** Quantitative analysis of AchR-positive per field at 2 weeks. **j** Quantitative analysis of AchR-positive per field at 4 weeks. **k** Frequency distribution of Feret’s diameter at 4 weeks. All data are presented as means ± SD, *n* = 4, *p < 0.05, **p < 0.01, ***p < 0.001 compared to NT, #*p* < 0.05, ##*p* < 0.01, ###*p* < 0.001 compared to CTR

In order to assess the level of vascularization in the VML, we employed immunofluorescence labeling on CD31 + cells and α-SMA + cells, since angiogenesis is a key component of tissue regeneration [72]. We discovered that the CTR and PTC groups had more CD31 + and α-SMA + cells and exhibited higher vascular density at 2 weeks post-surgery than the NT group. The CD31 + and α-SMA + areas all decreased at 4 weeks (Fig. 10a, b). The immunohistochemical results at 2 weeks and 4 weeks after surgery showed that the vascular density of the CTR and PTC groups was greater than that in the NT group. Among them, hydrogels in the PTC group had the best effect on angiogenesis, which may be related to the microstructures of the directional arrangement fibers of the hydrogel scaffold. Previous studies have proved that the orientated topography of biomaterials can promote the expression of angiogenesis-related gene and protein [73, 74]. The above results confirm that PTC hydrogel can promote vascular reconstruction during muscle tissue regeneration.

Nerve innervation is crucial for the long-term functional recovery of injured muscles [75]. Therefore, we evaluated nerve regeneration in the VML defect area in each group by labeling neuromuscular junction AchR. The results in Fig. 10c show that the PTC group had the highest expression level of AchR, followed by CTR, while the NT group had the worst expression level. This indicates that the directional nanomorphology of the hydrogel surface after mechanical training can significantly promote neural regeneration in the skeletal muscle defect area. Figure 10d shows the immunofluorescence images of the MHC-positive tissue. At 4 weeks post-surgery, the diameter of myofibers in the PTC group was significantly larger than in the CTR and NT groups, indicating a more mature structure (Fig. 10k).

## Conclusion

This work proposes a novel, versatile, and simple preparation method for tough hydrogel scaffolds: salting-out-assisted stretching training and photo-cross-linking treatment. This strengthening strategy can not only prepare scaffolds with the mechanical properties of bionic human tissue but is also applicable to a variety of hydrogel types. The gelatin-based tough hydrogel scaffold had a strength of 6.66 MPa, which is 622 times higher than that of the original hydrogel scaffold. In addition, this scaffold had good biocompatibility and could guide cell growth in a directional manner. After being implanted into animals, it could effectively promote muscle tissue regeneration within 4 weeks, increase muscle fiber production and blood vessel regeneration, and stimulate the tissue to quickly return to its original shape. This paper provides a novel, versatile strategy for preparing biological grade tough hydrogel scaffolds suitable for tissue engineering.

## Supplementary Information

Below is the link to the electronic supplementary material.

Supplementary file1 (DOCX 1351 kb)
