# Supplementary material for: 3D Printing of Tough Hydrogel Scaffolds with Functional Surface Structures for Tissue Regeneration
Source: Nanomicro Lett. 2024 Sep 29;17:27. doi: 10.1007/s40820-024-01524-z (PMC11439863; doi:10.1007/s40820-024-01524-z)
Supplement: Supplementary file 1 — Supplementary file1 (DOCX 1351 kb) [file 40820_2024_1524_MOESM1_ESM.docx]

Supporting Information for

**3D Printing of Tough Hydrogel Scaffolds with Functional Surface Structures for Tissue Regeneration**

Ke Yao^1, #^, Gaoying Hong^4, #^, Ximin Yuan^5^, Weicheng Kong^1^, Pengcheng Xia^6^, Yuanrong Li^1^, Yuewei Chen^1^, Nian Liu^1^, Jing He^1^, Jue Shi^4^, Zihe Hu^4^, Yanyan Zhou^4^, Zhijian Xie^4,^ * Yong He^1, 2, 3,^ *

^1^ State Key Laboratory of Fluid Power and Mechatronic Systems, School of Mechanical Engineering, Zhejiang University, Hangzhou 310027, P. R. China

^2^ Liangzhu Laboratory, Zhejiang University, Hangzhou 310027, P. R. China

^3^ Key Laboratory of 3D Printing Process and Equipment of Zhejiang Province, College of Mechanical Engineering, Zhejiang University, Hangzhou 310027, P. R. China

^4^ Stomatology Hospital, School of Stomatology, Zhejiang University School of Medicine, Hangzhou 310027, P. R. China

^5^ State Key Laboratory of Advanced Welding and Joining, Harbin Institute of Technology, Harbin 150001, P. R. China

^6^ Institute of Digital Medicine, Nanjing First Hospital, Nanjing Medical University, Nanjing 210006, P. R. China

^#^ Ke Yao and Gaoying Hong s contributed equally to this work.

*Corresponding authors. E-mail: [yongqin@zju.edu.cn](mailto:yongqin@zju.edu.cn) (Yong He); [xzj66@zju.edu.cn](file:///C:\Users\18702\AppData\Roaming\Microsoft\Word\xzj66@zju.edu.cn) (Zhijian Xie)

# Supplementary Figures


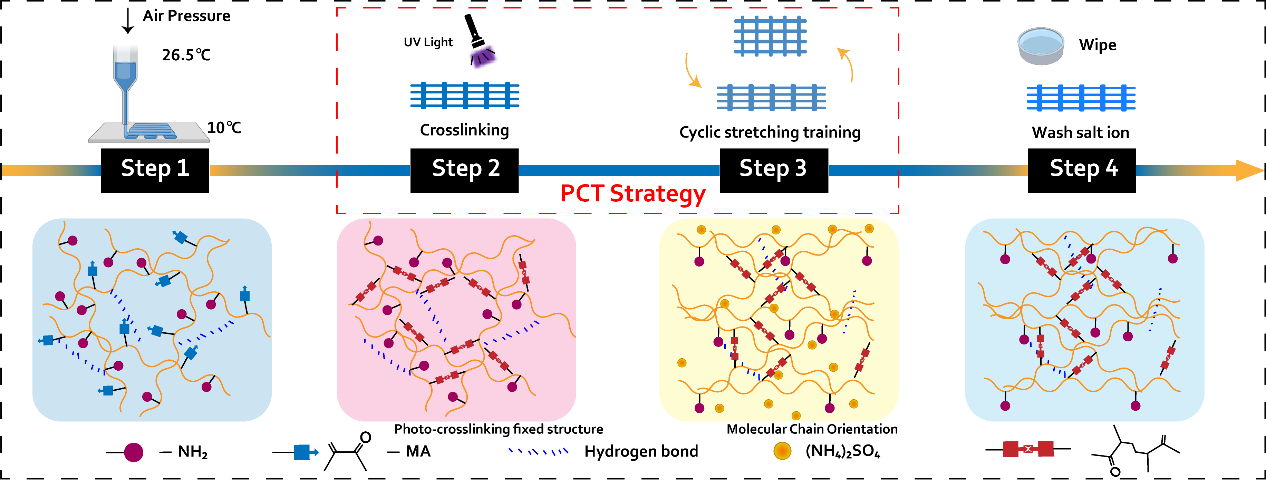


Fig. S1 Schematic diagram of PCT hydrogel preparation

The PCT manufacturing method is suitable for hydrogels that need to be photo-crosslinked first and then trained. Hydrogels trained by PCT also have extremely high strength and toughness, and have a certain directional structure at the microscopic level **(Fig. 1B)**.


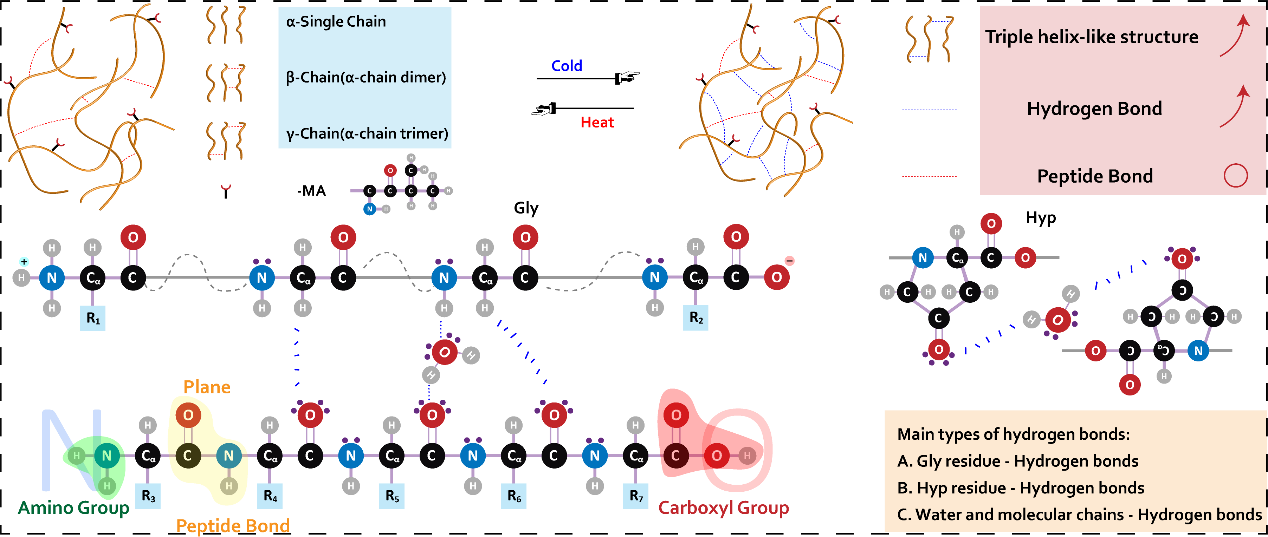


Fig. S2 Condensation changes and peptide chain and hydrogen bond composition during 3D printing process

The molecular chains of the GelMA precursor solution are scattered and disordered in the heated and melted state. After condensation, the triple helix structure increases, the number of hydrogen bonds increases, and it appears in a gel state. The hydrogen bonds in GelMA hydrogel are mainly Gly hydrogen bonds, Hyp hydrogen bonds and hydrogen bonds with water molecules.


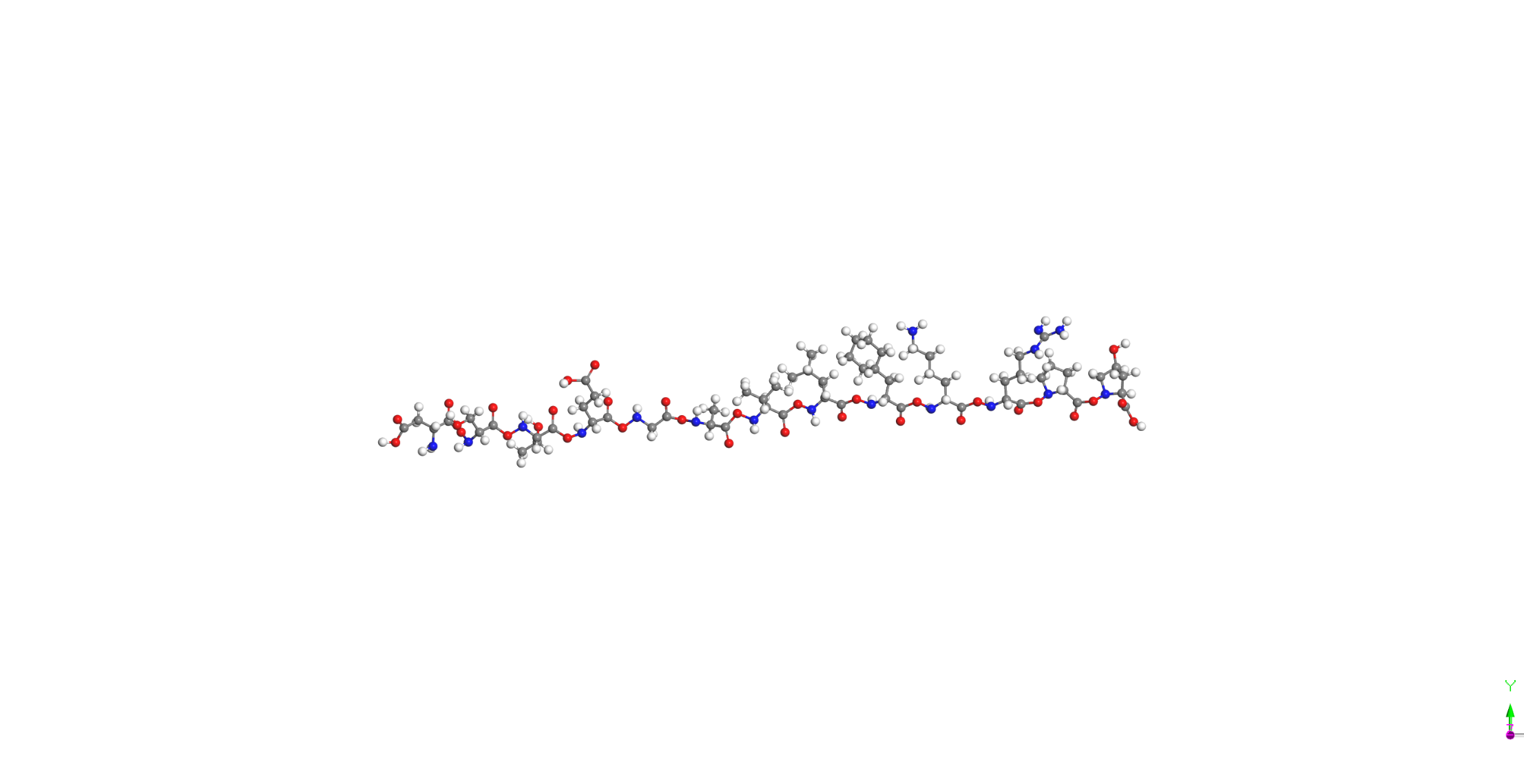

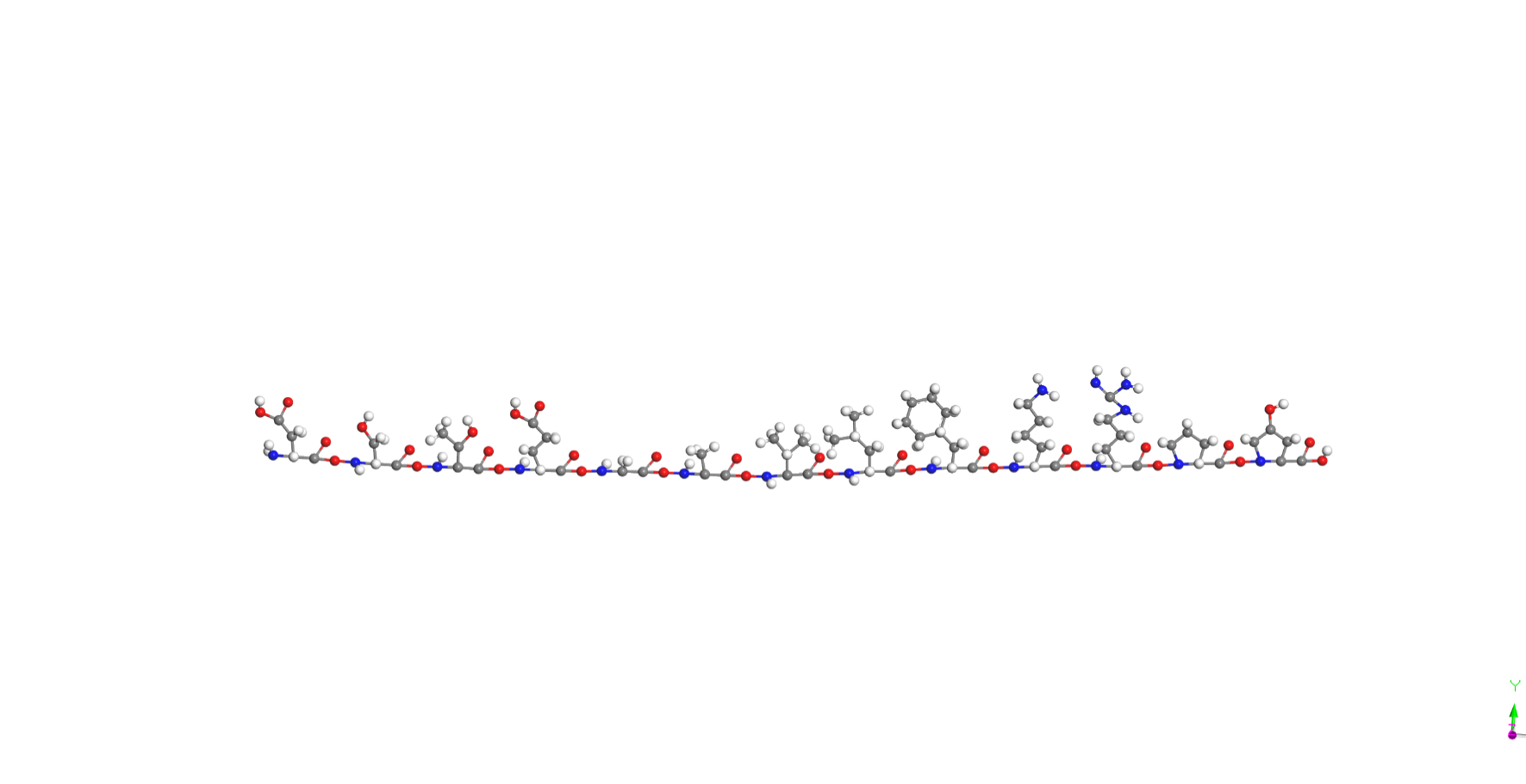


Fig. S3 Molecular dynamics GelMA modeling

The GelMA molecular chain model established in this article is modeled with 13 amino acids whose quantity is higher than 1%. The picture shows a straight long chain and a zigzag molecular chain.

The 13 amino acids are aspartic acid, threonine, serine, glutamic acid, glycine, alanine, valine, leucine, phenylalanine, lysine, arginine, and proline and hydroxyproline





Fig. S4 Energy contained in the electrons of sulfate radicals and water molecules

The ratio of the charges carried by sulfate radicals and water molecules when the abscissa is zero is used as the calculated value.


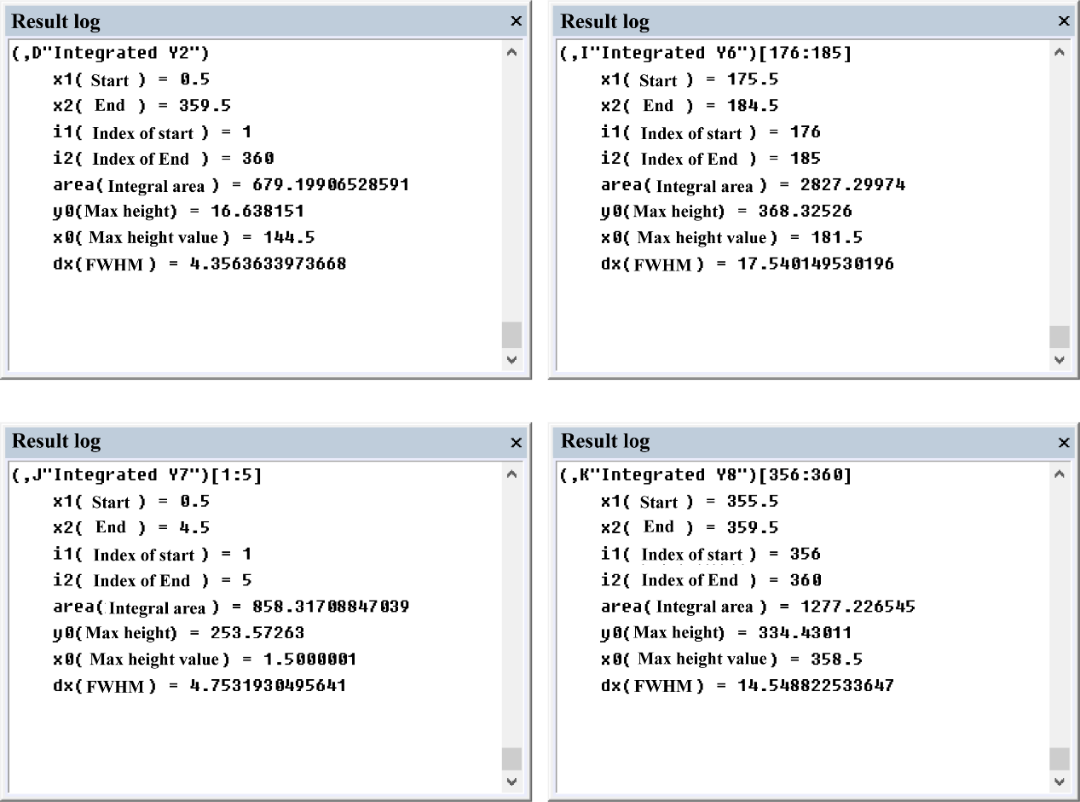


Fig. S5 SAXS detects the integral of two-dimensional azimuth angles

Integrating 2D azimuthal angles of initial and tough hydrogel scaffolds in SASX detection. The initial hydrogel scaffolds integration interval is 0°-360°. The tough hydrogel scaffolds integral intervals are 355°-5° and 175°-185°. Expressed as the influence rate of the degree of orientation before and after training on the strength of the hydrogel


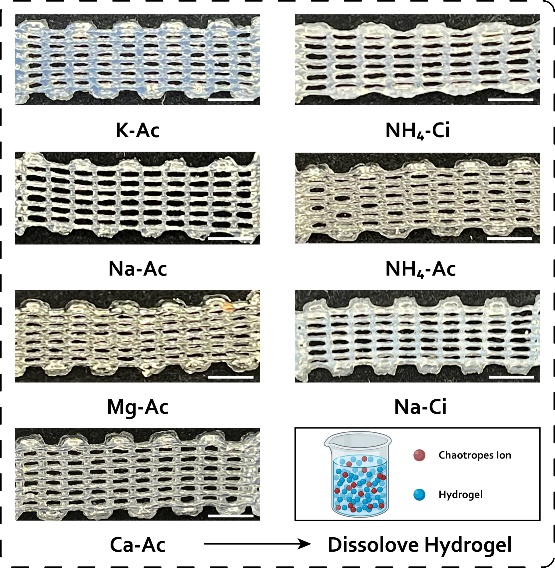


Fig. S7 Results of training tough hydrogel scaffolds in different types of salts, scale bar = 10mm

During the salting-out process, abundant hydrogen bonds are formed between hydrogels, and the GelMA hydrogel scaffold is strongly aggregated and partially crystallized, which comprise opaque crystalline aggregates that caused light to scatter, giving the hydrogel its white look. Magnesium salts and calcium salts are chaotropic salts. When the components of the saline solution during intensive training such salts, the hydrogel will soften or dissolve to varying degrees. During the training process, the chaotropic salt did not cause the GelMA hydrogel scaffold to produce opaque white crystalline areas, and the hydrogel remained transparent and colorless.


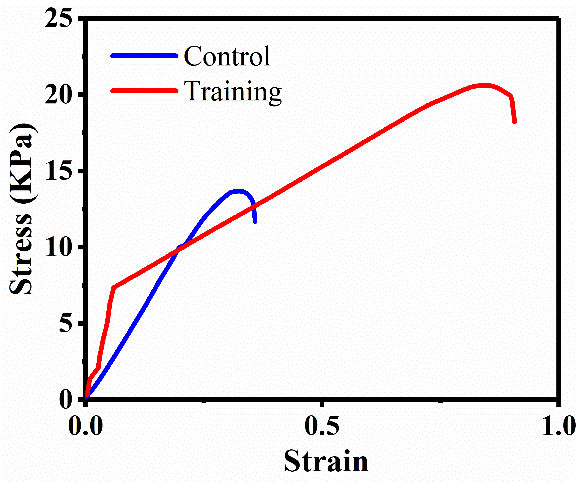

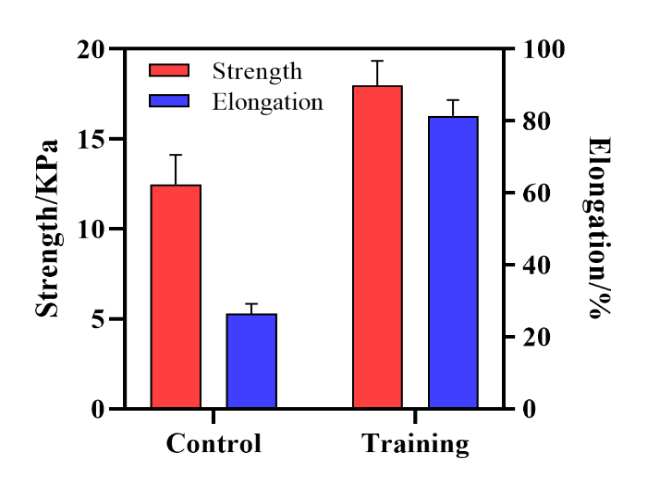


Fig. S8 Training results with HAMA hydrogel
